# Transcriptome sequencing and expression profiling of genes involved in the response to abiotic stress in *Medicago ruthenica*

**DOI:** 10.1590/1678-4685-GMB-2017-0284

**Published:** 2018-06-28

**Authors:** Yongjun Shu, Wei Li, Jinyue Zhao, Ying Liu, Changhong Guo

**Affiliations:** 1 College of Life Science and Technology, Harbin Normal University, Harbin Heilongjiang, China

**Keywords:** Medicago ruthenica, RNA-Seq, transcription factor (TF), abiotic stress, reactive oxygen species (ROS)

## Abstract

*Medicago ruthenica* is a perennial forage legume with the remarkable ability to survive under unfavorable environmental conditions. It has been identified as an excellent species of *Medicago* that can adapt to various environmental stresses including low temperature, drought, and salinity. To investigate its potential as a genetic resource, we performed transcriptome sequencing and analysis in *M. ruthenica* under abiotic stresses. We generated >120 million reads from six cDNA libraries, resulting in 79,249 unique transcripts, most of which were highly similar to transcripts from *M. truncatula* (44,608, 56.3%) and alfalfa (*M. sativa*, 48,023, 60.6%). Based on gene expression profiles, 2,721 transcripts were identified as abiotic stress responsive genes which were predicted to be mainly involved in phytohormone signaling pathways, transcriptional regulation, and ROS-scavenging. These results suggest that they play critical roles in the response to abiotic stress. In summary, we identified genes in our transcriptome dataset involved in the regulation of the abiotic stress response in *M. ruthenica* which will provide a valuable resource for the future identification and functional analysis of candidate genes for adaption to unfavorable conditions. The genes identified here could be also useful for improving stress tolerance traits in alfalfa through molecular breeding in the future.

## Introduction

In the natural environment, plant growth is often negatively affected by various unfavorable environmental conditions, such as temperature extremes, drought, and soil salinity. Unfavorable environments have selected for plants that can extensively modify their physiological and biochemical status to adapt to abiotic stresses, and these processes are controlled by complex regulatory networks involving numerous genes (Chinnusamy *et al.*, 2004). Based on their roles in response to stress, the genes are classified into two major functional groups: the first group is mainly comprised of genes encoding osmoprotectants, heat shock proteins (HSP), late embryogenesis abundant proteins (LEA) (Cuevas-Velazquez *et al.*, 2014), transporters (Dos Reis *et al.*, 2012), antioxidants (such as peroxidase, superoxide dismutase, and glutathione peroxidase) (Gill and Tuteja, 2010) and various kinds of metabolism-related proteins. These functional genes play important roles in protecting plants from the effects of environment stress. The second group is made up of genes involved in signal transduction and transcriptional regulation, a good example being transcription factors (TFs) that function by regulating the expression or status of other genes. At present, numerous TFs, such as members of the AP2/ERF, MYB, and NAC families, have been identified and characterized as being important regulators in the abiotic stress response in model plants (Golldack *et al.*, 2011; Chen *et al.*, 2012; Mizoi *et al.*, 2012).

*Medicago ruthenica* L. is an allogamous, diploid (2n=16) perennial legume forage crop that is widely distributed in Siberia, Mongolia, and northern China. Because of its high tolerance to various extreme environmental conditions, including low temperatures (cold and freezing), drought, and salinity (Campbell *et al.*, 1997; 1999), Balabaev (1934) noted that *M. ruthenica* has excellent prospects as a new forage species, and Campbell *et al.* (1999) positively evaluated its potential application in low input systems. Wang *et al.* (2008) hybridized *M. ruthenica* with *M. sativa*, and produced a new alfalfa cultivar (*M. sativa* cv. `Longmu No.3’) with high tolerance to freezing stress, which suggested that *M. ruthenica* can provide valuable genes for the genetic improvement of alfalfa through breeding. Both Yang *et al.* (2011) and Guan *et al.* (2009) investigated the physiological and biochemical responses of *M. ruthenica* to abiotic stresses, and their results have shown that this species is able to regulate its photosynthetic rate, stomatal conductance, and CO_2_ concentration to improve its tolerance to various stresses. However, *M. ruthenica* has received less attention in genetic research than did other *Medicago* species, such as *M. truncatula, M. falcata*, and *M. sativa* (alfalfa) (Pennycooke *et al.*, 2008), especially with respect to the molecular mechanisms underlying its tolerance to environmental stress.

Functional gene discovery and prediction have been mainly based on the sequencing of cloned single genes and EST sequencing, which are expensive and low throughput methods. Also, genome-wide gene expression levels are captured by microarray chip hybridization experiments, which are processed using an organism’s genetic information, and thus are limited in nonmodel organisms without gene sequences available for chip production. In the last decade, developments in next-generation sequencing (NGS) technologies have facilitated whole transcriptome sequencing, also known as RNA-Seq, which is widely used for revealing complex gene expression patterns in various organisms, including yeast, humans, Arabidopsis, and rice (Wang *et al.*, 2009; Ozsolak and Milos 2011). Recently, RNA-Seq has been increasingly used to identify and characterize genes that control important traits in livestock and crop plants, and it has proven to be a powerful tool for transcriptome analysis, particularly in nonmodel organisms for which a reference genome is not available (Strickler *et al.*, 2012).

In order to identify important genes that determine resistance to abiotic stress, we performed large-scale RNA-Seq of *M. ruthenica* under abiotic stresses, and transcriptome profiling allowed us to identify and characterize abiotic stress-responsive genes. The results of our study will provide novel insights into the response to abiotic stresses in *M. ruthenica*, and will also potentially contribute to the genetic improvement of alfalfa in the future.

## Material and Methods

### Plant material and stress treatments

Seeds of *Medicago ruthenica* (cv. `Zhilixing’) were the kind gift of Prof. Hong Li from the Heilongjiang Animal Science Institute in Heilongjiang Province, China. This cultivar grows well in Heilongjiang province, with high freezing tolerance. As previously described for *M. truncatula* (Shu *et al.*, 2015, 2016), the seeds of *M. ruthenica* were also germinated in the dark for 24 hours, and the seedlings were then transplanted into pots containing a soil-less mix (perlite and sand, 3:1 by volume). The *M. ruthenica* plants were then grown in a growth chamber (Conviron E15, Canada), and irrigated with 0.5X Hoagland’s solution every other day. The growing conditions were set as follows: 14 hour photoperiod, 18/24 °C (light/dark) temperature conditions, and relative humidity ranging from 60-80%. After eight weeks, the seedlings were randomly divided into six groups: (1) for the control group, seedlings were grown at normal conditions as described above; (2) for the cold stress group, the seedlings were transferred to another chamber with the temperature set at 4 °C; (3) for the freezing group, the temperature was -8 °C; (4) for the osmotic stress group, the seedlings were treated with 300 mM mannitol solution; (5) for the salt stress group, the seedlings were treated with 200 mM NaCl solution; and (6) for the ABA treatment group, the seedling leaves were sprayed with 100 μM ABA solution. All seedlings were harvested 3 h after treatment. For each treatment group, five whole seedlings were randomly selected and separately bulked, flash frozen in liquid nitrogen, and stored at -80 °C prior to use in the experiments.

### RNA sequencing library construction and high-throughput sequencing

Total RNA was extracted from seedlings in the six treatment groups using the RNeasy Plant Mini Kit (Qiagen, Valencia, CA, USA) following the manufacturer’s instructions. Total RNAs extracted from the six samples was then quantified using a NanoDrop 2000 spectrophotometer (Thermo Fisher, Waltham, USA) and an Agilent 2100 Bioanalyzer (Agilent Technologies, Santa Clara, USA). The transcriptome sequencing libraries were constructed by BGI-Shenzhen Co. Ltd (Shenzhen, China) as previously described (Shu *et al.*, 2015). Nucleotide sequencing was performed on the Illumina GAII platform, and 100 bp paired-end reads were generated.

### *De novo* assembly and functional annotation

The raw Illumina sequencing reads were cleaned by removing adapter sequences, PCR duplication reads, empty reads, and reads with low quality scores (Q<20). The clean reads from the three libraries were then combined, and Trinity software was used for *de novo* transcriptome assembly with the parameters “min_kmer_cov 2” (Haas *et al.*, 2013). Considering that there is redundancy in the *de novo* assembly results, we used iAssembler (Zheng *et al.*, 2011) and CD-HIT-EST clusters (Li and Godzik 2006) for further contig assembly. The resulting unique sequences were identified as *M. ruthenica* unigene transcripts.

To investigate the genetic relationships with *M. truncatula* and *M. sativa* (alfalfa), we used these transcripts as queries in BLASTN searches against *M. truncatula* (http://www.medicagogenome.org/; Young *et al.*, 2011) and alfalfa transcript (http://plantgrn.noble.org/AGED; O’Rourke *et al.*, 2015) databases with an E-value threshold of 1E-30. The *M. ruthenica* transcripts, with identities of >90%, were identified as homologs to *M. truncatula* or alfalfa transcripts. For functional annotation of the *M. ruthenica* transcripts, we used BLASTP searches against *M. truncatula,* soybean, and Arabidopsis protein sequences with an E-value of 1E-5, and functional protein annotations, including Gene ontology (GO), and KOG (Eu**K**aryotic **O**rthologous **G**roups) annotations, were assigned to the matching *M. ruthenica* transcripts. The GO annotation results were viewed using the online tool WEGO (http://wego.genomics.org.cn) (Ye *et al.*, 2006). To explore regulatory roles of transcription factors in the *M. ruthenica* response to abiotic stresses, these transcripts were analyzed using the iTAK pipeline (http://bioinfo.bti.cornell.edu/tool/itak) (Jin *et al.*, 2014) for plant transcription factor identification and classification, and their respective family members were also evaluated.

### Identification of differentially-expressed transcripts

The clean reads from the six RNA-Seq libraries were mapped to the assembled *M. ruthenica* transcripts using TopHat (Trapnell *et al.*, 2009), and the FPKM values (fragments per kilobase of exon per million fragments mapped) for all transcripts were evaluated with Cufflinks software (Trapnell *et al.*, 2012). Each treatment group was then compared with the control group using R platform packages, and transcripts with fold changes ≥2 or ≤0.5 and an adjusted *p*-value ≤0.05 were identified as being differentially expressed.

The GO annotation results of these differentially expressed transcripts in the response to abiotic stresses were retrieved, and GO functional enrichment analyses were performed using the topGO package in R (Robinson *et al.*, 2010). The degree of enrichment in each GO term was called the rich factor, which was calculated as follows: [rich factor = ((number of transcripts differentially expressed in test GO term)/(number of transcripts differentially expressed with GO annotation))/((number of transcripts in test GO term)/(number of all transcripts with GO annotation)].

In order to compare genes in the response to abiotic stresses in the *Medicago* genus, transcriptome data from *M. truncatula*, *M. sativa*, and *M. falcata* (Miao *et al.*, 2015) was also downloaded, and we performed RNA-Seq analysis using this data as described above.

## Results

### Transcriptome assembly and annotation in *M. ruthenica*

**Figure 1:**
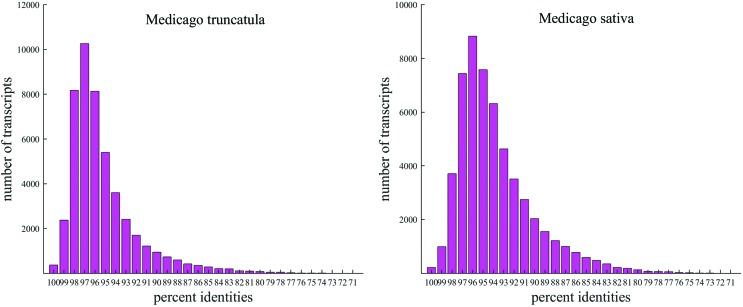
Sequence identity distributions of unique *Medicago ruthenica* transcripts against *M. truncatula* and *M. sativa* (alfalfa). The X-axes show the percent similarity of *Medicago ruthenica* transcripts to the two other *Medicago* species, and the Y-axes show the numbers of transcripts. The results of this analysis show that transcribed genes from *M. ruthenica* are highly similar to genes from both *M. truncatula* and alfalfa.

Using high-throughput Illumina DNA sequencing, >120 million reads were generated from six cDNA libraries. Among the raw reads, low quality reads, those containing adapter sequences, or low quality bases were discarded, and the remaining clean reads were deposited in the NCBI SRA database (Accession numbers: SRR4140266, 68-72). These clean reads were then assembled *de novo* using Trinity software, and redundant transcripts were eliminated using iAssemble and CD-HIT-EST. A total of 79,249 assembled transcripts were generated from the six *M. ruthenica* cDNA libraries, and they were all identified as *M. ruthenica* genes. Mean gene length was 1,020 bp, with an N50 value of 1,673 bp (details are shown in Table 1). The *M. ruthenica* transcripts had the highest sequence identity with *M. truncatula* and alfalfa, (44,608 and 48,023 transcripts were homologous with *M. truncatula* and alfalfa, respectively; Figure 1). As shown from BLAST search results, 51,115 (64.5%) of the assembled transcripts were identified with significant hits to combinations of proteins from *M. truncatula*, soybean, and Arabidopsis.

**Table 1:** Summary of assembly statistics for the *Medicago ruthenica* transcriptome.

Data type	Number
Total sequence	79,249
Number of sequences in 201-500 bp	32,901
Number of sequences in 500-1000 bp	16,750
Number of sequences more than 1000 bp	29,598
Minimal length (bp)	201
Maximal length (bp)	1,1741
N50 (bp)	1,673
Average length (bp)	1,020

**Figure 2:**
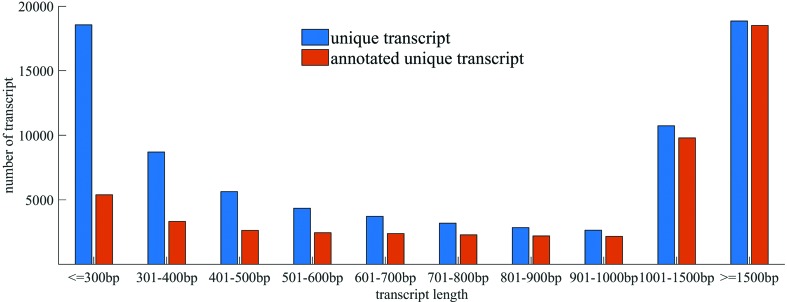
Length distribution of unique *Medicago ruthenica* transcripts. Transcript length is shown on the X-axis, and the Y-axis shows the number of transcripts. The numbers and relative proportions of annotated transcripts increase with transcript length, implying that longer transcripts have a higher probability of being annotated.

**Figure 3:**
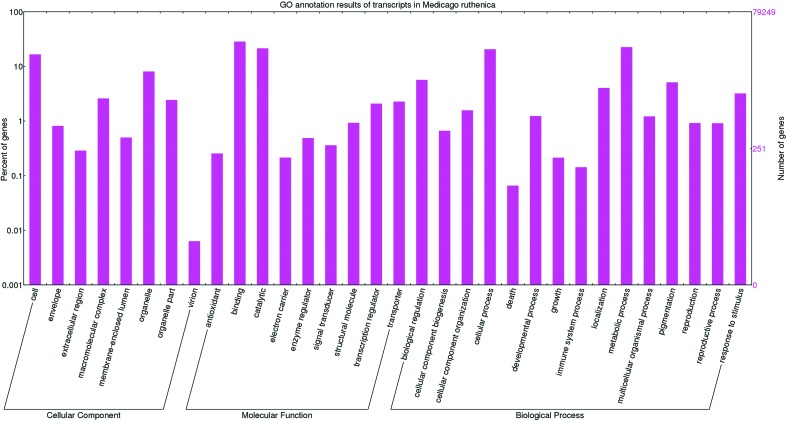
GO classification results for annotated unique transcripts in *Medicago ruthenica*. The *Medicago ruthenica* transcripts were annotated using BLASTP searches against transcriptome databases for *M. truncatula*, soybean, and Arabidopsis, and the results were categorized and viewed using WEGO. Percentage of genes (y-axis) indicates the proportion of Medicago ruthenica unique transcripts that have relevant GO annotations in the three major GO domains “cellular components”, “molecular function”, and “biological process”.

It is worthy of note that longer unigenes had more hits than did the short ones (Figure 2), implying that a percentage of the unigene annotations was positively correlated with sequence length, which is consistent with previous reports. Among these unigenes, 45,338 (57.2%) were assigned at least one GO term from the three main domains “biological process”, “molecular function”, and “cellular component” (Figure 3). Most genes in the biological process category were mainly classified into the groups “cellular process” (GO:0009987), “metabolic process” (GO:0008152), “biological regulation” (GO:0065007), and “response to stimulus” (GO:0050896). In the “molecular function” category, genes were focused on terms including binding (GO:0005488), catalytic activity (GO:0003824), transporter activity (GO:0005215) and transcription regulator activity (GO:0030528). There were also numerous genes present in specific GO terms; for example, antioxidant activity (GO:0016209), which shows that antioxidants play an important role in the response to abiotic stresses, which has also been reported in other plants.

**Figure 4:**
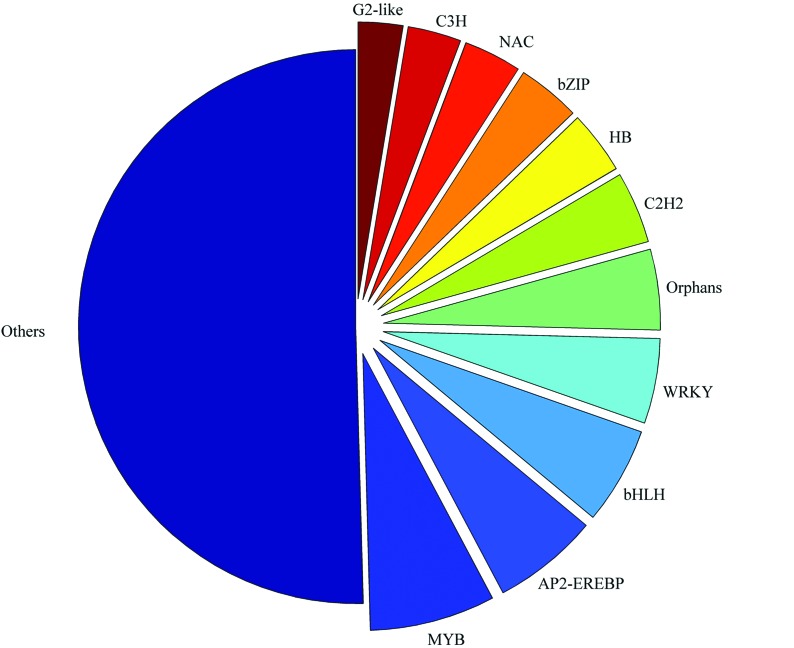
Distribution of transcription factor genes in the differentially expressed *Medicago ruthenica* transcriptome sequences. The transcription factor genes were identified and classified using iTAK. The pie plot shows that MYB, AP2/ERF, bHLH, and WRKY are the largest TF family genes expressed in the *Medicago ruthenica* abiotic stress response.

To find genes involved in the transcription regulation process, these genes were scanned using the iTAK pipeline for identifying transcription factors. In total, 1,953 TFs classified in 79 families were identified from the *M. ruthenica* unigenes (Figure 4). The majority of the *M. ruthenica* TFs were found to be members of the MYB, AP2/ERF, bHLH, and WRKY families, which have been previously shown to play important roles in the abiotic stress response in model plant species such as Arabidopsis, rice, and soybean.

### Identification and characterization of abiotic stress response genes in *M. ruthenica*

**Figure 5:**
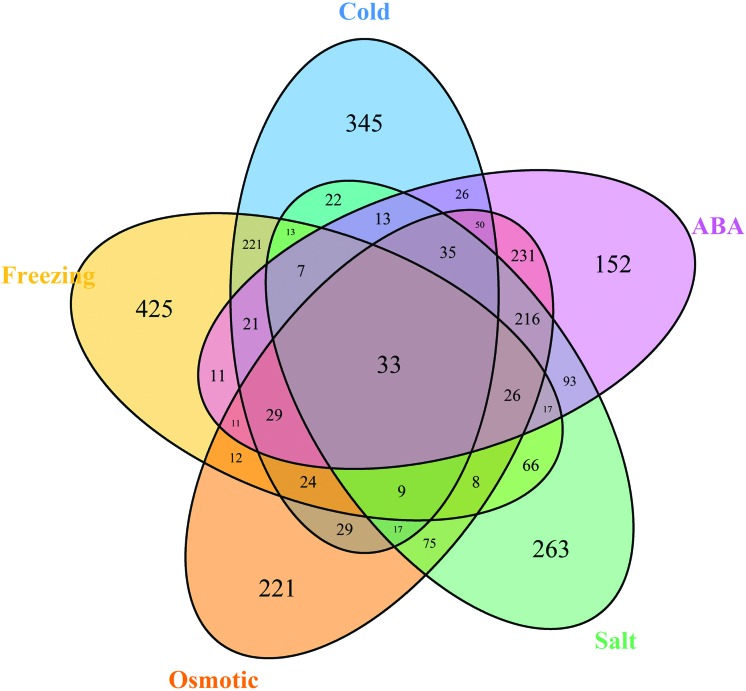
Diagrammatic representation of the distributions of *Medicago ruthenica* genes that are differentially expressed in response to five abiotic stresses. The Venn diagram shows the overlap of DEGs in the responses to various abiotic stresses. The cold and freezing stress groups share more commonly expressed genes, while the osmotic, salt and ABA stress groups probably contain other DEGs.

To determine which genes play a role in the abiotic stress response in *M. ruthenica*, five comparisons between the control group and the abiotic stress groups (cold, freezing, osmotic, salt, and ABA), were performed. We identified 2,721 differentially expressed genes with a false discovery rate of 0.05. Specifically, there were 894 genes that responded to cold stress, 933 to freezing stress, 1,026 to osmotic stress, 913 to salt stress, and 971 to ABA treatment (Figure 5). Among these genes, 33 showed differential expression across all five abiotic stress treatments, implying that these genes are commonly expressed in *M. ruthenica* in response to abiotic stress, and also that their functions are highly enriched in transcription factors (GO analysis results, four genes), indicating that transcriptional regulation is the main means of conferring abiotic stress tolerance. Other genes were specifically either induced or repressed by an individual stress treatment; for example, 345 genes in cold stress, 425 genes in freezing stress, 221 genes in osmotic stress, 263 genes in salt stress, and 152 genes in response to ABA treatment. In addition, a considerable number of genes were simultaneously affected by two stress treatments; for example, 221 genes were differentially expressed in response to both cold and freezing, implying that these two stresses potentially share common regulatory pathways. Similarly, there were 231 genes that responded to both osmotic stress and ABA treatment, and 216 that responded to salt and ABA, which were also suggests that ABA plays important roles in the responses to both osmotic and salt stress. To determine their genetic function in the abiotic stress response, KOG we performed annotation analysis (Figure S1). The results showed that the DEGs involved in abiotic stress could be classified into diverse categories, such as signal transduction, amino acid transport and metabolism, and carbohydrate transport and metabolism, implying that *M. ruthenica* probably employs different genetic regulation pathways to confer tolerance to various abiotic stresses.

### Functional annotation and enrichment analysis of the DEGs

**Figure 6:**
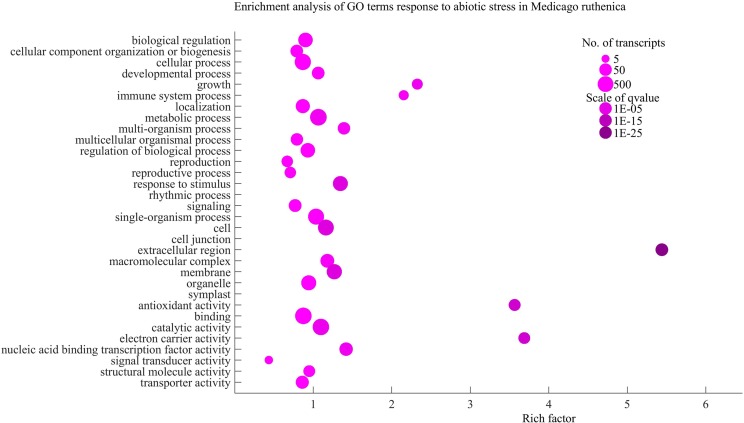
Gene ontology (GO) enrichment analysis of *Medicago ruthenica* unique transcripts response to abiotic stress. The enriched transcripts are plotted in the various GO terms as pink circles, and the sizes of the circles indicate the relative number of transcripts present in each GO term. The color scale is based on *p*-value. Dark pink circles are the most significantly over-represented, while light pink circles represent the least significant terms.

To investigate the functions of these DEGs, we performed GO annotation enrichment analysis using the software package topGO. We found that the DEGs were significantly enriched in 235 GO terms (Table S1). In the “biological processes” category, the main terms, including growth (GO:0040007), immune system process (GO:0002376), photosynthesis (GO:0015979), and response to stimuli (GO:0050896), were highly enriched in the DEGs, suggesting that genes involved in these processes potentially play important roles in the responses to abiotic stresses. In the “molecular function” category, DEGs were significantly enriched in the two terms nucleic acid binding transcription factor activity (GO:0001071) and antioxidant activity (GO:0016209) (Figure 6), implying that TFs and antioxidants have critical functions in the plant response to abiotic stress, which has been shown in other plants. For example, TFs regulate the expression of down-stream functional genes for adapting to stress, while antioxidants protect plant cells from oxidative damage by scavenging reactive oxygen species (ROS). In total, 160 TF genes were identified that responded to one or more specific stress (Figure S2), many of which have been previously shown to have important roles in the abiotic stress response, such as members of the AP2/ERF, bHLH, MYB, WRKY, and NAC TF families (Table S2). Antioxidants, such as peroxidases, ascorbate, and glutathione, eliminate ROS generated by various stresses, protecting plants from intracellular oxidative damage that can occur during stress.

**Figure 7:**
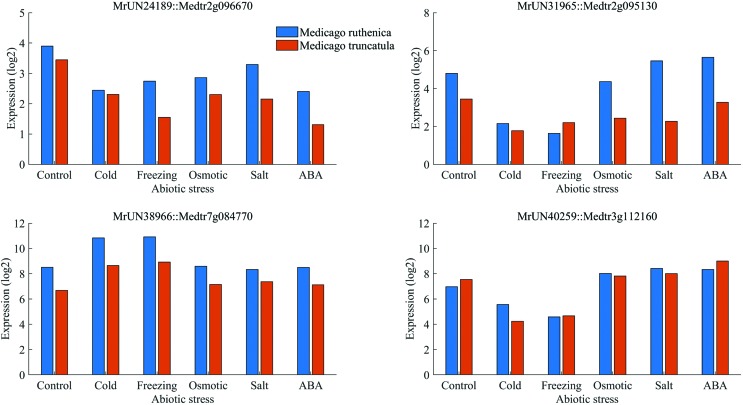
Comparisons of the expression profiles of four *Medicago ruthenica* unigenes that are differentially expressed in the response to abiotic stress and their homologs in *M. truncatula*. Four pairs of homologous genes from *Medicago ruthenica* and *M. truncatula* show similar expression profiles in control plants and plants exposed to cold, freezing, osmotic, salt, and ABA stress treatments. The expression levels were calculated using FPKM values.

Similar transcriptome analyses were also performed in the model plant *M. truncatula*, and 3,360 DEGs were identified (the sequences were downloaded from the NCBI SRA database under accession numbers SRX1056987-92). It is worthy of note that we identified 698 *M. ruthenica* abiotic stress DEGs in which the homologs were also differentially expressed in *M. truncatula* (Table S3). We performed 1,000 computer simulations, using 2,721 randomly selected genes from *M. ruthenica* and 3,360 genes from *M. truncatula* in each simulation, and the mean number of genes present in both the *M. ruthenica* and *M. truncatula* gene sampling list (homologous gene pairs) was 109, which was much smaller than the number of common genes in the response to abiotic stresses, implying that the expression patterns and functions of many genes are highly conserved between the two species (Figure 7). In addition, functional annotation analysis of these genes showed that they are highly enriched in stress-related processes, including photosynthesis (GO:0015979), oxidation-reduction processes (GO:0055114), and response to stress (GO:0006950) (Table S4).

We also analyzed transcriptome sequences from other *Medicago* species, including *M. sativa* and *M. falcata*. All DEGs were mapped to the *M. truncatula* transcript database for multi-species comparison, and the results showed that *M. ruthenica* and *M. falcata* are both important resources for the genetic improvement of alfalfa in response to abiotic stress (Figure S3).

## Discussion

In the present study we performed an RNA-Seq analysis of the *M. ruthenica* transcriptome in response to five different abiotic stresses using high-throughput nucleotide sequencing. In total, >120M reads were obtained, and 79,249 unigenes were generated by *de novo* assembly of the pooled RNA-Seq reads. The average length of the assembled unigenes was 1,020 bp, and the N50 value was 1,673 bp, indicating that the assembly was of high quality, and implying that many of the unigenes contained full-length coding regions. In addition, 60.6% of the unigenes (48,023 unigenes) had high similarities (≥90%) with alfalfa transcripts; considering that it is highly possible that *M. ruthenica* could hybridize with alfalfa, these unigenes could be an important resource for alfalfa genetic improvement, especially with regards to abiotic stress tolerance. The unigenes involved in the response to abiotic stress are discussed below.

### Phytohormones that regulate the response to abiotic stress

Phytohormones, including abscisic acid (ABA), auxins, gibberellins (GA), cytokinins (CK), ethylene (ET), jasmonates (JA), and brassinosteroids (BR), have long been recognized as the key plant hormones that mediate plant growth, development, and responses to abiotic stress (Kumar 2013; Verma *et al.*, 2016). It is now known that these phytohormones extensively regulate all aspects of plant stress responses, ranging from signal cascade transduction to modifications in plant developmental processes.

Generally, ABA is the best-studied phytohormone with respect to the plant stress response, and it regulates downstream gene expression through the ABA-responsive element (ABRE) (Sah *et al.*, 2016). Many transcription factors and functional genes have been identified as ABA target genes in various stresses; examples are TFs from the MYB, NAC, and DREB families. In our study, 16 unigenes involved in the response to ABA were identified as being differentially expressed, which supports the important function of ABA in response to abiotic stress (Table S5). Among these regulatory genes, the expression of MrUN03429 and MrUN27218, which both contain a BURP domain, responded to various stresses, which was consistent with previous reports showing the important roles of these genes in abiotic stress (Li *et al.*, 2016). GA has been shown to regulate plant growth to withstand stress damage, and we have identified GA-regulated unigenes involved in the cell elongation and division processes, including expansin (MrUN07876), GASA/GAST/Snakin (MrUN19564, MrUN21275, MrUN21674, MrUN27354, and MrUN39273), implying their roles in the stress response via modification of plant growth (Colebrook *et al.*, 2014). Similarly, DEGs were identified and characterized for auxins; several unigenes belonging to the SAUR gene family were identified as being responsive to stress, and these are well known to be regulated by auxins in response to various stresses in many plants (Ren and Gray, 2015).

### Transcription factors involved in abiotic stress responses

Many transcription factors (TFs) have been characterized that play important roles in plant responses to abiotic stresses such as cold, freezing, osmotic stress, and salt. In the current study, we identified 160 TFs that were differentially expressed in the response to abiotic stress in the *M. ruthenica* transcriptome. Most of these TFs were identified as members of several TF families known to function in plant responses, including AP2/ERF, bHLH, MYB, WRKY, C2H2, and NAC. The largest was the AP2/ERF TF family, members of which have been widely characterized for their roles in cold, osmotic, and salt stress from numerous plants, including Arabidopsis, rice, maize, and soybean. In the present study, we identified 26 AP2/ERF TF genes that showed differential expression in response to abiotic stress, similar to our previous finding in *M. truncatula* (Shu *et al.*, 2015). This result implies that the critical function of these genes in the abiotic stress response is highly conserved in plants, and that they have a potential application in alfalfa genetics and breeding. In addition, genes from other TF families with important roles in abiotic stress responses have been identified in other plants.

In the MYB family, AtMYB2 has been shown to be induced by dehydration and salt stress (Urao *et al.*, 1996), AtMYB96 was reported to be involved in the response to drought stress (Seo *et al.*, 2009), OsMYB3R-2 was characterized and demonstrated to improve freezing, drought, and salt stress tolerance in transgenic plants (Dai *et al.*, 2007), and three soybean MYB genes (GmMYB72, GmMYB96 and GmMYB117) were also shown to be regulated by cold, drought, salt, and and/or ABA stresses (Liao *et al.*, 2008). In our study, were found that 14 MYB TF genes were regulated by various stresses; for example, expression of MrUN10866, MrUN33504, MrUN37588, and MrUN40182 was induced by cold and/or freezing stress, while MrUN28786 was up-regulated by all stresses. These results confirm previous reports in other plants, and confirmed that MYB TFs have positive functions in the response to abiotic stresses. Similar results were confirmed for genes in other well characterized abiotic stress responsive TF families, such as WRKY (Chen *et al.*, 2012) and NAC (Shao *et al.*, 2015).

### ROS as key players in abiotic stress responses

In the plant response to abiotic stress, oxidative stress becomes an important secondary stress due to the accumulation of intracellular reactive oxygen species (ROS), which are harmful for plant growth by destroying cellular components and resulting in programmed cell death (Sewelam *et al.*, 2016). To protect plant seedlings from oxidative damage, plants have established a complex physiological system to scavenge ROS (Baxter *et al.*, 2014; Del Rio and Lopez-Huertas, 2016).

In the present study, we identified numerous genes involved in ROS-scavenging systems that were significantly regulated by abiotic stress based on MapMan annotation results (Usadel *et al.*, 2009). In the *M. ruthenica* transcriptome, we identified 90 peroxidase (POD) genes, and 17 of these gene were identified as being up- or down-regulated by RNA-Seq, implying that they have a protective function against oxidative damage under abiotic stress conditions. In addition, members of other familiar gene families of ROS-scavenging systems have also been shown wo be differentially expressed in response to abiotic stresses (Baxter *et al.*, 2014; Zinta *et al.*, 2016); examples are glutathione (GSHs, two members), glutathione S transferase (GSTs, nine members), peroxiredoxins (PRXs, two members) and glutaredoxins (GRXs, six members). However, the details of ROS-scavenging systems are still largely unknown, and they they are worthy of future study.

## Conclusion

In this study, a transcriptome dataset of genes expressed in *M. ruthenica* in response to five abiotic stresses was generated by high-throughput Illumina RNA sequencing. The RNA-Seq results generated a total of 79,249 assembled transcripts, and 2,721 of these transcripts were identified as abiotic stress responsive genes that are mainly involved in transcriptional regulation, phytohormone signaling pathways, and ROS scavenging. These findings were helpful in exploring the *M. ruthenica* response to abiotic stress, and important genes could be candidates for introduction into alfalfa with a high potential to improve abiotic stress tolerance in the future.

## Supplementary material

Tthe following online material is available for this article:

Figure S1 - KOG functional classification of *Medicago ruthenica* differentially expressed transcripts.

Figure S2 - Diagrammatic representation of the distributions of differentially expressed transcripts.

Figure S3 - Heatmap showing the expression profiles of transcription factor genes.

Table S1 - Functional enrichment analysis of GO terms using topGO for differentially expressed genes.

Table S2 - Numbers of DEGs identified as transcription factor genes.

Table S3 - Common DEG pairs identified in both the *Medicago ruthenica* and *Medicago truncatula* responses to abiotic stress.

Table S4 - Functional enrichment analysis of GO terms.

Table S5 - Phytohormone-related unigenes that were differentially expressed.
