# Supplementary material for: Transcriptome sequencing and expression profiling of genes involved in the response to abiotic stress in Medicago ruthenica
Source: Genet Mol Biol. 2018 Jun 28;41(3):638–48. doi: 10.1590/1678-4685-GMB-2017-0284 (PMC6136363; doi:10.1590/1678-4685-GMB-2017-0284)
Supplement: Supplementary file 1 [file 1415-4757-GMB-1678-4685-GMB-2017-0284-s001.pdf]

Supplementary Material to "Transcriptome sequencing and expression profiling of genes involved in the response to abiotic stress in *Medicago ruthenica*"

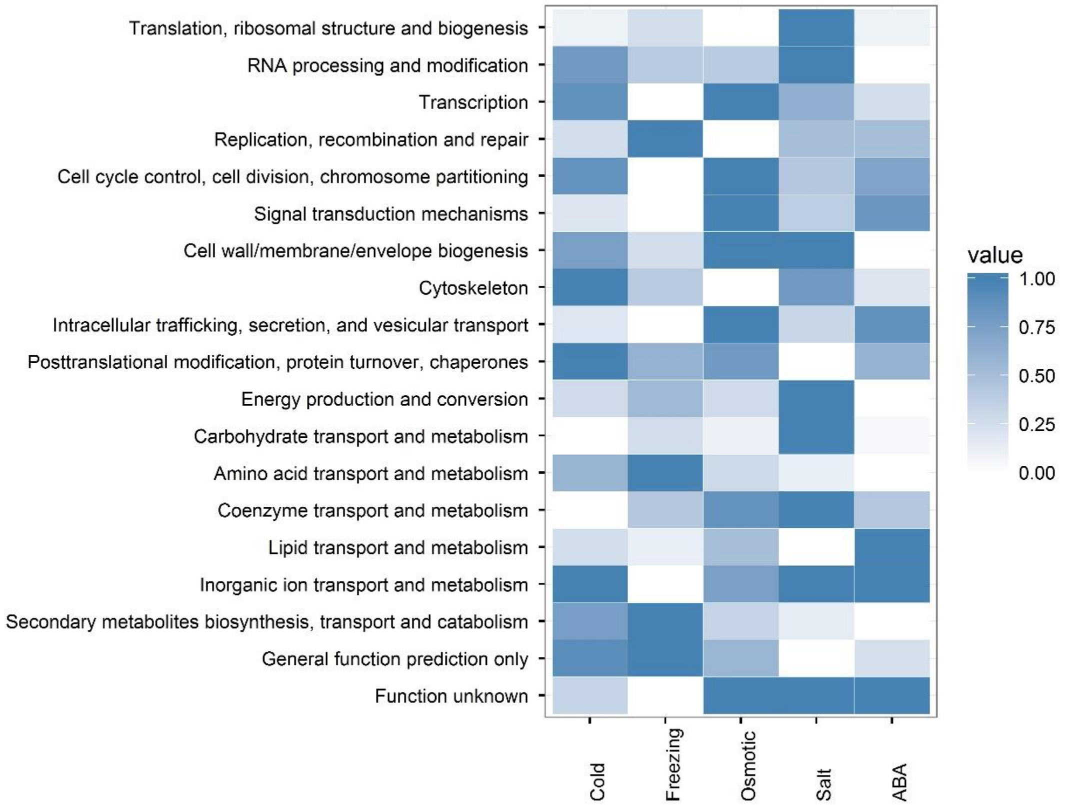

Figure S1 - KOG functional classification of *Medicago ruthenica* differentially expressed transcripts in the responses to five different abiotic stress treatments. The value of each KOG term is based on the numbers of transcripts present in KOG term; 0 (white) indicates that no transcripts are present, or the fewest transcripts are present, and a value of 1 (dark blue) indicates the highest number of transcripts present.
