# Supplementary material for: Transcriptome sequencing and expression profiling of genes involved in the response to abiotic stress in Medicago ruthenica
Source: Genet Mol Biol. 2018 Jun 28;41(3):638–48. doi: 10.1590/1678-4685-GMB-2017-0284 (PMC6136363; doi:10.1590/1678-4685-GMB-2017-0284)
Supplement: Supplementary file 2 [file 1415-4757-GMB-1678-4685-GMB-2017-0284-s004.pdf]

Supplementary Material to "Transcriptome sequencing and expression profiling of genes involved in the response to abiotic stress in *Medicago ruthenica*"

Table S1 - Functional enrichment analysis of GO terms using topGO for differentially expressed genes in the *Medicago ruthenica* response to abiotic stress

Group	GO Terms	Term Description	Total Transcripts	Significant Transcripts	P-value
	GO:0016491	oxidoreductase activity	2767	247	8.50E-21
	GO:0046906	tetrapyrrole binding	650	91	1.60E-19
	GO:0020037	heme binding	621	79	9.10E-15
	GO:0016762	xyloglucan:xyloglucosyl transferase acti...	63	20	9.50E-12
	GO:0016209	antioxidant activity	207	36	3.20E-11
	GO:0016758	transferase activity, transferring hexos...	771	82	4.00E-11
	GO:0009055	electron carrier activity	189	34	4.30E-11
	GO:0004553	hydrolase activity, hydrolyzing O-glycos...	822	84	1.70E-10
	GO:0004601	peroxidase activity	183	32	3.40E-10
	GO:0016684	oxidoreductase activity, acting on perox...	183	32	3.40E-10
	GO:0016705	oxidoreductase activity, acting on paire...	854	83	2.40E-09
	GO:0016798	hydrolase activity, acting on glycosyl b...	883	85	2.40E-09
	GO:0016757	transferase activity, transferring glyco...	1001	93	2.60E-09
	GO:0016984	ribulose-bisphosphate carboxylase activi...	9	7	2.10E-08
	GO:0015035	protein disulfide oxidoreductase activit...	61	16	2.40E-08
	GO:0015036	disulfide oxidoreductase activity	61	16	2.40E-08
**MF**	GO:0016168	chlorophyll binding	28	11	3.60E-08
	GO:0008171	O-methyltransferase activity	78	17	1.70E-07
	GO:0005509	calcium ion binding	373	43	1.90E-07
	GO:0005506	iron ion binding	528	54	2.90E-07
	GO:0005507	copper ion binding	184	26	1.10E-06
	GO:0046872	metal ion binding	3817	249	1.20E-06
	GO:0004857	enzyme inhibitor activity	151	23	1.20E-06
	GO:0043169	cation binding	3839	250	1.30E-06
	GO:0045735	nutrient reservoir activity	82	15	9.10E-06
	GO:0016667	oxidoreductase activity, acting on a sul...	112	17	3.10E-05
	GO:0051213	dioxygenase activity	403	39	4.30E-05
	GO:0016706	oxidoreductase activity, acting on paire...	307	32	5.00E-05
	GO:0004066	asparagine synthase (glutamine-hydrolyzi...	10	5	5.60E-05
	GO:0030599	pectinesterase activity	96	15	6.30E-05
	GO:0010277	chlorophyllide a oxygenase [overall] act...	6	4	7.80E-05
	GO:0003824	catalytic activity	16955	907	0.00011

Group	GO Terms	Term Description	Total Transcripts	Significant Transcripts	P-value
	GO:0016746	transferase activity, transferring acyl ...	544	46	0.00024
	GO:0001071	nucleic acid binding transcription facto...	1372	95	0.00042
	GO:0003700	sequence-specific DNA binding transcript...	1372	95	0.00042
	GO:0000990	core RNA polymerase binding transcriptio...	15	5	0.00055
	GO:0000996	core DNA-dependent RNA polymerase bindin...	15	5	0.00055
	GO:0016987	sigma factor activity	15	5	0.00055
	GO:0016160	amylase activity	32	7	0.00075
	GO:0051537	2 iron, 2 sulfur cluster binding	16	5	0.00076
	GO:0052689	carboxylic ester hydrolase activity	227	23	0.00079
	GO:0008061	chitin binding	10	4	0.00094
	GO:0016851	magnesium chelatase activity	10	4	0.00094
	GO:0051002	ligase activity, forming nitrogen-metal ...	10	4	0.00094
	GO:0051003	ligase activity, forming nitrogen-metal ...	10	4	0.00094
	GO:0003854	3-beta-hydroxy-delta5-steroid dehydrogen...	37	7	0.00185
	GO:0016229	steroid dehydrogenase activity	37	7	0.00185
	GO:0033764	steroid dehydrogenase activity, acting o...	37	7	0.00185
	GO:0004650	polygalacturonase activity	71	10	0.00228
	GO:0045156	electron transporter, transferring elect...	2	2	0.00238
	GO:0016703	oxidoreductase activity, acting on singl...	13	4	0.00283
	GO:0046933	proton-transporting ATP synthase activit...	14	4	0.00381
	GO:0004866	endopeptidase inhibitor activity	65	9	0.00417
	GO:0030414	peptidase inhibitor activity	65	9	0.00417
	GO:0061134	peptidase regulator activity	65	9	0.00417
	GO:0061135	endopeptidase regulator activity	65	9	0.00417
	GO:0016830	carbon-carbon lyase activity	143	15	0.00435
	GO:0008289	lipid binding	290	25	0.00445
	GO:0004332	fructose-bisphosphate aldolase activity	8	3	0.0054
	GO:0051536	iron-sulfur cluster binding	106	12	0.00558
	GO:0051540	metal cluster binding	106	12	0.00558
	GO:0004867	serine-type endopeptidase inhibitor acti...	27	5	0.00904
	GO:0016831	carboxy-lyase activity	100	11	0.00958
	GO:0019829	cation-transporting ATPase activity	18	4	0.00997
	GO:0044769	ATPase activity, coupled to transmembran...	18	4	0.00997
	GO:0015979	photosynthesis	148	68	< 1e-30
	GO:0055114	oxidation-reduction process	2442	232	2.10E-18
	GO:0019684	photosynthesis, light reaction	58	26	2.90E-18
BP	GO:0006979	response to oxidative stress	282	52	4.20E-15
	GO:0006950	response to stress	1697	164	8.80E-14
	GO:0009628	response to abiotic stimulus	830	95	2.30E-12
	GO:0009765	photosynthesis, light harvesting	30	15	5.60E-12

Group	GO Terms	Term Description	Total Transcripts	Significant Transcripts	P-value
	GO:1901700	response to oxygen-containing compound	423	59	1.70E-11
	GO:0009416	response to light stimulus	312	48	4.30E-11
	GO:0010114	response to red light	29	14	5.30E-11
	GO:0006952	defense response	328	49	7.70E-11
	GO:0009314	response to radiation	326	48	2.00E-10
	GO:0009767	photosynthetic electron transport chain	17	10	2.50E-09
	GO:0009607	response to biotic stimulus	352	48	2.70E-09
	GO:0006091	generation of precursor metabolites and ...	240	36	2.20E-08
	GO:0006869	lipid transport	87	20	2.40E-08
	GO:0010876	lipid localization	87	20	2.40E-08
	GO:0009644	response to high light intensity	64	17	2.70E-08
	GO:0009405	pathogenesis	9	7	4.00E-08
	GO:0009637	response to blue light	32	12	4.30E-08
	GO:0010033	response to organic substance	625	67	5.40E-08
	GO:0050896	response to stimulus	3992	287	5.80E-08
	GO:0042221	response to chemical	964	91	1.10E-07
	GO:0001101	response to acid	316	41	1.50E-07
	GO:0006073	cellular glucan metabolic process	205	31	1.70E-07
	GO:0044042	glucan metabolic process	205	31	1.70E-07
	GO:0009642	response to light intensity	72	17	1.80E-07
	GO:0015977	carbon fixation	25	10	2.90E-07
	GO:0009773	photosynthetic electron transport in pho...	11	7	3.40E-07
	GO:0005976	polysaccharide metabolic process	267	35	9.40E-07
	GO:0010218	response to far red light	41	12	9.70E-07
	GO:0009719	response to endogenous stimulus	511	54	1.60E-06
	GO:0009266	response to temperature stimulus	281	35	3.10E-06
	GO:0009725	response to hormone	490	51	4.70E-06
	GO:0044264	cellular polysaccharide metabolic proces...	241	31	5.70E-06
	GO:0009768	photosynthesis, light harvesting in phot...	4	4	8.10E-06
	GO:0009639	response to red or far red light	121	19	2.30E-05
	GO:0071555	cell wall organization	166	23	2.70E-05
	GO:0009617	response to bacterium	135	20	3.40E-05
	GO:0098542	defense response to other organism	157	22	3.40E-05
	GO:0009611	response to wounding	96	16	4.70E-05
	GO:0045229	external encapsulating structure organiz...	172	23	4.80E-05
	GO:0005975	carbohydrate metabolic process	1522	117	4.80E-05
	GO:0042542	response to hydrogen peroxide	50	11	5.30E-05
	GO:0045454	cell redox homeostasis	162	22	5.50E-05
	GO:0042545	cell wall modification	129	19	5.70E-05
	GO:0010035	response to inorganic substance	311	34	6.60E-05
	GO:0006528	asparagine metabolic process	10	5	8.70E-05
	GO:0006529	asparagine biosynthetic process	10	5	8.70E-05
	GO:0019725	cellular homeostasis	179	23	8.90E-05

Group	GO Terms	Term Description	Total Transcripts	Significant Transcripts	P-value
	GO:0080167	response to karrikin	82	14	0.00011
	GO:0009408	response to heat	147	20	0.00011
	GO:0033993	response to lipid	194	24	0.00012
	GO:0071554	cell wall organization or biogenesis	257	29	0.00013
	GO:0043207	response to external biotic stimulus	309	33	0.00013
	GO:0051707	response to other organism	309	33	0.00013
	GO:0022900	electron transport chain	55	11	0.00013
	GO:0050832	defense response to fungus	55	11	0.00013
	GO:0009664	plant-type cell wall organization	96	15	0.00017
	GO:0009739	response to gibberellin	39	9	0.00017
	GO:0048589	developmental growth	108	16	0.0002
	GO:0009828	plant-type cell wall loosening	49	10	0.00022
	GO:0009827	plant-type cell wall modification	59	11	0.00026
	GO:0040007	growth	169	21	0.00028
	GO:0000302	response to reactive oxygen species	80	13	0.00031
	GO:0014070	response to organic cyclic compound	113	16	0.00034
	GO:0044710	single-organism metabolic process	5672	356	0.00039
	GO:0009620	response to fungus	63	11	0.00046
	GO:0009817	defense response to fungus, incompatible...	20	6	0.00047
	GO:0071669	plant-type cell wall organization or bio...	107	15	0.00057
	GO:0042742	defense response to bacterium	96	14	0.00057
	GO:0060560	developmental growth involved in morphog...	98	14	0.00071
	GO:0009409	response to cold	145	18	0.00075
	GO:0009826	unidimensional cell growth	89	13	0.00087
	GO:0044262	cellular carbohydrate metabolic process	400	37	0.00089
	GO:0008152	metabolic process	17713	1008	0.00096
	GO:0009831	plant-type cell wall modification involv...	31	7	0.00104
	GO:0042547	cell wall modification involved in multi...	31	7	0.00104
	GO:0016049	cell growth	151	18	0.00121
	GO:0015837	amine transport	5	3	0.0014
	GO:0032890	regulation of organic acid transport	5	3	0.0014
	GO:0032973	amino acid export	5	3	0.0014
	GO:0044070	regulation of anion transport	5	3	0.0014
	GO:0051952	regulation of amine transport	5	3	0.0014
	GO:0051955	regulation of amino acid transport	5	3	0.0014
	GO:0080143	regulation of amino acid export	5	3	0.0014
	GO:0010019	chloroplast-nucleus signaling pathway	2	2	0.00285
	GO:0018149	peptide cross-linking	2	2	0.00285
	GO:0018316	peptide cross-linking via L-cystine	2	2	0.00285
	GO:0019253	reductive pentose-phosphate cycle	2	2	0.00285
	GO:0019685	photosynthesis, dark reaction	2	2	0.00285
	GO:0009744	response to sucrose	20	5	0.00342

Group	GO Terms	Term Description	Total Transcripts	Significant Transcripts	P-value
	GO:0034285	response to disaccharide	20	5	0.00342
	GO:0009651	response to salt stress	219	22	0.00344
	GO:0010583	response to cyclopentenone	58	9	0.00346
	GO:0051607	defense response to virus	13	4	0.00393
	GO:0009699	phenylpropanoid biosynthetic process	21	5	0.00429
	GO:0009806	lignan metabolic process	21	5	0.00429
	GO:0009807	lignan biosynthetic process	21	5	0.00429
	GO:0015995	chlorophyll biosynthetic process	21	5	0.00429
	GO:0071229	cellular response to acid	71	10	0.00435
	GO:0006949	syncytium formation	30	6	0.00453
	GO:0015994	chlorophyll metabolic process	40	7	0.00485
	GO:0006694	steroid biosynthetic process	42	7	0.0064
	GO:0010206	photosystem II repair	8	3	0.00696
	GO:0030091	protein repair	8	3	0.00696
	GO:0042592	homeostatic process	290	26	0.00716
	GO:0002376	immune system process	113	13	0.00735
	GO:0009615	response to virus	77	10	0.00775
	GO:0006970	response to osmotic stress	236	22	0.00822
	GO:0009772	photosynthetic electron transport in pho...	3	2	0.00825
	GO:0009733	response to auxin	181	18	0.00845
	GO:0015985	energy coupled proton transport, down el...	34	6	0.00855
	GO:0015986	ATP synthesis coupled proton transport	34	6	0.00855
	GO:0097305	response to alcohol	169	17	0.00923
	GO:0009415	response to water	117	13	0.00975
	GO:0009579	thylakoid	358	112	< 1e-30
	GO:0034357	photosynthetic membrane	300	103	< 1e-30
	GO:0044436	thylakoid part	330	107	< 1e-30
	GO:0042651	thylakoid membrane	262	82	< 1e-30
	GO:0009521	photosystem	88	45	< 1e-30
	GO:0009535	chloroplast thylakoid membrane	207	63	< 1e-30
	GO:0055035	plastid thylakoid membrane	207	63	< 1e-30
	GO:0009534	chloroplast thylakoid	237	66	< 1e-30
	GO:0031976	plastid thylakoid	237	66	< 1e-30
**CC**	GO:0031984	organelle subcompartment	237	66	< 1e-30
	GO:0044434	chloroplast part	603	99	1.60E-27
	GO:0005576	extracellular region	221	58	4.40E-27
	GO:0044435	plastid part	630	100	1.30E-26
	GO:0009523	photosystem II	55	30	1.90E-25
	GO:0048046	apoplast	128	41	4.20E-23
	GO:0005618	cell wall	355	59	5.40E-17
	GO:0030312	external encapsulating structure	355	59	5.40E-17
	GO:0009507	chloroplast	2129	186	1.40E-16
	GO:0009570	chloroplast stroma	226	45	3.00E-16

Group	GO Terms	Term Description	Total Transcripts	Significant Transcripts	P-value
	GO:0009532	plastid stroma	255	48	3.20E-16
	GO:0009536	plastid	2187	186	2.10E-15
	GO:0009654	photosystem II oxygen evolving complex	35	15	2.10E-11
	GO:0009522	photosystem I	30	14	2.40E-11
	GO:0031977	thylakoid lumen	57	16	6.80E-09
	GO:0019898	extrinsic component of membrane	50	15	7.30E-09
	GO:0009538	photosystem I reaction center	6	6	1.20E-08
	GO:0009573	chloroplast ribulose bisphosphate carbox...	9	7	2.00E-08
	GO:0048492	ribulose bisphosphate carboxylase comple...	9	7	2.00E-08
	GO:0016020	membrane	5642	345	5.60E-08
	GO:0005623	cell	9800	549	6.30E-08
	GO:0044464	cell part	9800	549	6.30E-08
	GO:0030076	light-harvesting complex	22	9	3.80E-07
	GO:0009505	plant-type cell wall	81	16	1.30E-06
	GO:0009941	chloroplast envelope	303	33	1.10E-05
	GO:1990204	oxidoreductase complex	96	16	1.30E-05
	GO:0031225	anchored component of membrane	129	19	1.40E-05
	GO:0009543	chloroplast thylakoid lumen	41	10	1.90E-05
	GO:0031978	plastid thylakoid lumen	41	10	1.90E-05
	GO:0071944	cell periphery	1496	107	2.20E-05
	GO:0030095	chloroplast photosystem II	5	4	2.60E-05
	GO:0009526	plastid envelope	333	33	7.50E-05
	GO:0010287	plastoglobule	33	8	0.00013
	GO:0031012	extracellular matrix	21	6	0.00036
	GO:0044425	membrane part	2145	136	0.00045
	GO:0044422	organelle part	1845	119	0.00057
	GO:0044446	intracellular organelle part	1845	119	0.00057
	GO:0010598	NAD(P)H dehydrogenase complex (plastoqui...	5	3	0.00104
	GO:0009782	photosystem I antenna complex	2	2	0.00233
	GO:0000322	storage vacuole	7	3	0.00338
	GO:0000326	protein storage vacuole	7	3	0.00338
	GO:0043234	protein complex	1435	91	0.00418
	GO:0022626	cytosolic ribosome	91	11	0.00432
	GO:0000325	plant-type vacuole	15	4	0.00479
