# Supplementary material for: Transcriptome sequencing and expression profiling of genes involved in the response to abiotic stress in Medicago ruthenica
Source: Genet Mol Biol. 2018 Jun 28;41(3):638–48. doi: 10.1590/1678-4685-GMB-2017-0284 (PMC6136363; doi:10.1590/1678-4685-GMB-2017-0284)
Supplement: Supplementary file 3 [file 1415-4757-GMB-1678-4685-GMB-2017-0284-s002.pdf]

Supplementary Material to "Transcriptome sequencing and expression profiling of genes involved in the response to abiotic stress in *Medicago ruthenica*"

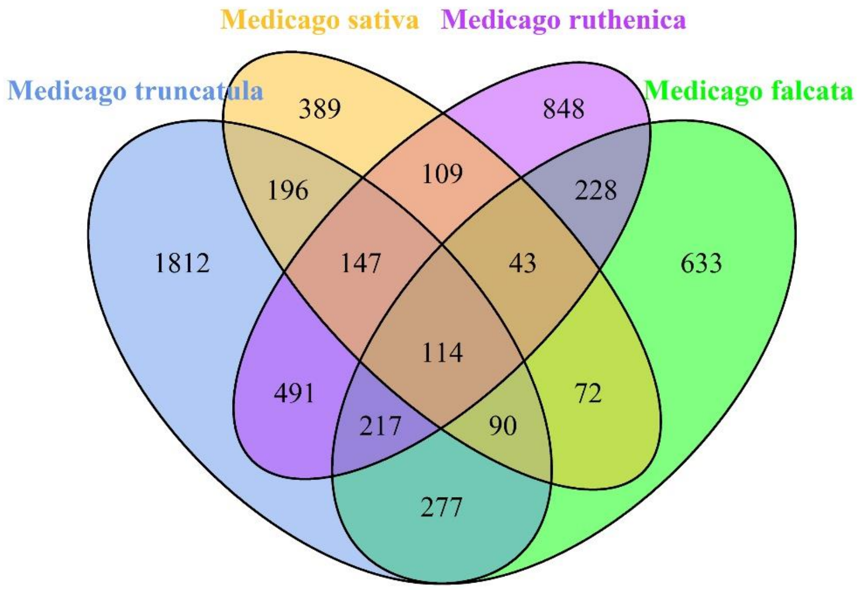

Figure S2 - Diagrammatic representation of the distributions of differentially expressed transcripts in the response to abiotic stresses in *Medicago*. The Venn diagram shows the overlap of differentially expressed genes in the response to various abiotic stresses in four species of *Medicago*. The *M. truncatula* and *M. sativa* RNA-Seq data were generated from our previous studies, and the *M. falcata* RNA-Seq data is from Miao *et al*. (2016). All DEGs were aligned to *M. truncatula* for determining their relationships, and the overlaps are shown based on their homology.
