# Supplementary material for: Transcriptome sequencing and expression profiling of genes involved in the response to abiotic stress in Medicago ruthenica
Source: Genet Mol Biol. 2018 Jun 28;41(3):638–48. doi: 10.1590/1678-4685-GMB-2017-0284 (PMC6136363; doi:10.1590/1678-4685-GMB-2017-0284)
Supplement: Supplementary file 4 [file 1415-4757-GMB-1678-4685-GMB-2017-0284-s005.pdf]

Supplementary Material to "Transcriptome sequencing and expression profiling of genes involved in the response to abiotic stress in *Medicago ruthenica*"

Table S2 - Numbers of DEGs identified as transcription factor genes in the *Medicago ruthenica* response to abiotic stress

TF family	Cold	Freezing	Osmotic	Salt	ABA	Total
AP2/ERF	6	10	15	9	13	26
bHLH	5	3	6	4	5	16
MYB	6	3	5	3	3	14
WRKY	7	4	12	4	9	14
C2H2	2	1	7	3	8	11
NAC	5	1	3	1	3	8
Tify	5	1	3	3	3	7
GRAS	0	0	2	0	4	5
Other	23	21	22	16	17	59
