# Supplementary material for: Transcriptome sequencing and expression profiling of genes involved in the response to abiotic stress in Medicago ruthenica
Source: Genet Mol Biol. 2018 Jun 28;41(3):638–48. doi: 10.1590/1678-4685-GMB-2017-0284 (PMC6136363; doi:10.1590/1678-4685-GMB-2017-0284)
Supplement: Supplementary file 5 [file 1415-4757-GMB-1678-4685-GMB-2017-0284-s006.pdf]

Supplementary Material to "Transcriptome sequencing and expression profiling of genes involved in the response to abiotic stress in *Medicago ruthenica*"

Table S3 - Common DEG pairs identified in both the *Medicago ruthenica* and *Medicago truncatula* responses to abiotic stress

Medicago ruthenica transcripts	*Medicago truncatula* genes
MrUN00466	Medtr1g078110
MrUN00602	Medtr2g103170
MrUN00610	Medtr3g100500
MrUN00763	Medtr5g090630
MrUN01215	Medtr4g102670
MrUN01257	Medtr4g076470
MrUN01274	Medtr3g083310
MrUN01289	Medtr4g078710
MrUN01299	Medtr1g084200
MrUN01395	Medtr2g103490
MrUN01483	Medtr5g022870
MrUN01524	Medtr4g099260
MrUN01662	Medtr5g010830
MrUN01757	Medtr3g007240
MrUN01923	Medtr3g115050
MrUN01940	Medtr7g088880
MrUN01966	Medtr2g096210
MrUN01967	Medtr2g435490
MrUN01980	Medtr8g059170
MrUN02207	Medtr6g051780
MrUN02241	Medtr4g075333
MrUN02276	Medtr1g062590
MrUN02342	Medtr1g069805
MrUN02363	Medtr1g101600
MrUN02438	Medtr2g099910
MrUN02444	Medtr6g032820
MrUN02450	Medtr2g028940
MrUN02488	Medtr7g095170
MrUN02580	Medtr1g087920
MrUN02607	Medtr1g090060
MrUN02644	Medtr2g013570
MrUN02674	Medtr4g114870

Medicago ruthenica transcripts	*Medicago truncatula* genes
MrUN02697	Medtr7g085260
MrUN02975	Medtr2g067980
MrUN02982	Medtr6g014270
MrUN03079	Medtr7g081570
MrUN03100	Medtr5g017670
MrUN03103	Medtr3g051770
MrUN03181	Medtr5g047060
MrUN03229	Medtr7g076900
MrUN03253	Medtr5g022210
MrUN03331	Medtr7g106440
MrUN03422	Medtr3g462220
MrUN03452	Medtr3g077550
MrUN03533	Medtr2g087810
MrUN03609	Medtr4g124040
MrUN03674	Medtr8g018800
MrUN03752	Medtr3g011820
MrUN03836	Medtr3g104560
MrUN03864	Medtr1g066530
MrUN04419	Medtr8g027495
MrUN04508	Medtr4g105740
MrUN04725	Medtr1g069450
MrUN04737	Medtr4g075710
MrUN04771	Medtr4g086240
MrUN04843	Medtr6g034060
MrUN04887	Medtr4g055420
MrUN04930	Medtr3g068150
MrUN04953	Medtr1g018760
MrUN05033	Medtr1g069605
MrUN05118	Medtr8g096900
MrUN05232	Medtr4g108940
MrUN05493	Medtr1g021652
MrUN05496	Medtr1g103090
MrUN05525	Medtr1g054450
MrUN05577	Medtr1g097935
MrUN05684	Medtr5g025750
MrUN05738	Medtr3g098650
MrUN05838	Medtr7g102490
MrUN05992	Medtr5g075100
MrUN06009	Medtr5g063740
MrUN06010	Medtr8g028425
MrUN06175	Medtr1g052880
MrUN06244	Medtr0055s0050
MrUN06248	Medtr2g084000
MrUN06254	Medtr8g104190

Medicago ruthenica transcripts	*Medicago truncatula* genes
MrUN06368	Medtr1g019360
MrUN06369	Medtr3g114750
MrUN06382	Medtr8g081040
MrUN06386	Medtr7g095930
MrUN06393	Medtr3g086940
MrUN06395	Medtr8g099185
MrUN06419	Medtr4g067150
MrUN06439	Medtr4g035430
MrUN06494	Medtr8g078240
MrUN06542	Medtr7g022440
MrUN06552	Medtr6g082280
MrUN06565	Medtr3g106250
MrUN06617	Medtr2g049780
MrUN06688	Medtr4g053250
MrUN06752	Medtr8g096310
MrUN06782	Medtr5g019650
MrUN06832	Medtr4g013385
MrUN06906	Medtr0015s0140
MrUN07015	Medtr1g115560
MrUN07138	Medtr1g072420
MrUN07143	Medtr4g007710
MrUN07182	Medtr1g060550
MrUN07217	Medtr6g014187
MrUN07272	Medtr2g013310
MrUN07307	Medtr4g127120
MrUN07311	Medtr3g083580
MrUN07352	Medtr4g095310
MrUN07403	Medtr2g064840
MrUN07409	Medtr6g008210
MrUN07422	Medtr1g056870
MrUN07465	Medtr4g082330
MrUN07615	Medtr6g016015
MrUN07722	Medtr3g103460
MrUN07746	Medtr8g079950
MrUN07758	Medtr8g106680
MrUN07876	Medtr7g011060
MrUN07922	Medtr2g041960
MrUN07925	Medtr3g072610
MrUN07939	Medtr8g095360
MrUN08034	Medtr5g013530
MrUN08045	Medtr1g033840
MrUN08098	Medtr5g035980
MrUN08141	Medtr4g046713
MrUN08252	Medtr4g019580

Medicago ruthenica transcripts	*Medicago truncatula* genes
MrUN08265	Medtr0003s0660
MrUN08267	Medtr0003s0660
MrUN08273	Medtr7g068110
MrUN08294	Medtr4g088770
MrUN08472	Medtr5g072400
MrUN08507	Medtr8g027860
MrUN08522	Medtr4g037690
MrUN08662	Medtr4g094492
MrUN08679	Medtr0334s0010
MrUN08761	Medtr3g110172
MrUN08846	Medtr5g027810
MrUN08959	Medtr1g026410
MrUN09122	Medtr4g105750
MrUN09211	Medtr1g102860
MrUN09382	Medtr2g102140
MrUN09487	Medtr5g012600
MrUN09554	Medtr7g111880
MrUN09590	Medtr6g012380
MrUN09678	Medtr1g058490
MrUN09861	Medtr4g112460
MrUN09984	Medtr4g087830
MrUN10130	Medtr5g036080
MrUN10174	Medtr2g013010
MrUN10212	Medtr8g012240
MrUN10680	Medtr8g024790
MrUN10796	Medtr1g105360
MrUN10804	Medtr8g103700
MrUN11053	Medtr3g080800
MrUN11060	Medtr7g115650
MrUN11144	Medtr1g026380
MrUN11158	Medtr5g029190
MrUN11225	Medtr3g065250
MrUN11300	Medtr1g105495
MrUN11338	Medtr3g064070
MrUN11553	Medtr4g013770
MrUN11733	Medtr5g021670
MrUN11761	Medtr2g009980
MrUN12021	Medtr8g075960
MrUN12028	Medtr1g090370
MrUN12038	Medtr3g091440
MrUN12105	Medtr4g128580
MrUN12160	Medtr4g106970
MrUN12220	Medtr4g063090
MrUN12236	Medtr4g049340

Medicago ruthenica transcripts	*Medicago truncatula* genes
MrUN12298	Medtr3g073180
MrUN12346	Medtr2g076070
MrUN12358	Medtr3g062930
MrUN12366	Medtr1g075640
MrUN13133	Medtr1g112510
MrUN13203	Medtr7g102380
MrUN13322	Medtr7g104460
MrUN13345	Medtr3g074860
MrUN13383	Medtr1g115410
MrUN13478	Medtr3g110205
MrUN13512	Medtr4g068970
MrUN13763	Medtr5g014260
MrUN13812	Medtr0365s0010
MrUN13926	Medtr3g073140
MrUN13989	Medtr2g090765
MrUN14033	Medtr2g101380
MrUN14094	Medtr5g089820
MrUN14140	Medtr6g008480
MrUN14333	Medtr4g015860
MrUN14417	Medtr3g435320
MrUN14497	Medtr4g109470
MrUN15098	Medtr5g019920
MrUN17822	Medtr4g051290
MrUN18705	Medtr6g471080
MrUN18987	Medtr3g008470
MrUN19129	Medtr3g008920
MrUN19133	Medtr3g072520
MrUN19155	Medtr2g038040
MrUN19166	Medtr4g134290
MrUN19167	Medtr5g040430
MrUN19210	Medtr7g068650
MrUN19215	Medtr4g092690
MrUN19238	Medtr2g088080
MrUN19253	Medtr8g081600
MrUN19314	Medtr1g074990
MrUN19318	Medtr7g069980
MrUN19368	Medtr1g030600
MrUN19370	Medtr1g081660
MrUN19373	Medtr1g110570
MrUN19521	Medtr5g048050
MrUN19547	Medtr7g095220
MrUN19619	Medtr3g013500
MrUN19697	Medtr8g012420
MrUN19713	Medtr1g021760

Medicago ruthenica transcripts	*Medicago truncatula* genes
MrUN19714	Medtr2g064310
MrUN19730	Medtr1g077890
MrUN19874	Medtr2g020240
MrUN19875	Medtr4g080350
MrUN19956	Medtr3g008840
MrUN19960	Medtr4g132020
MrUN19962	Medtr1g105020
MrUN19968	Medtr1g492820
MrUN19971	Medtr5g063670
MrUN20028	Medtr4g059730
MrUN20061	Medtr2g102370
MrUN20072	Medtr3g461480
MrUN20113	Medtr4g011720
MrUN20115	Medtr4g011690
MrUN20173	Medtr5g040960
MrUN20191	Medtr5g026850
MrUN20264	Medtr6g066240
MrUN20265	Medtr2g076070
MrUN20338	Medtr5g085580
MrUN20378	Medtr4g094610
MrUN20445	Medtr1g040875
MrUN20456	Medtr0102s0060
MrUN20547	Medtr8g098945
MrUN20568	Medtr1g059660
MrUN20578	Medtr1g063350
MrUN20613	Medtr4g062330
MrUN20622	Medtr4g014460
MrUN20644	Medtr4g092820
MrUN20683	Medtr8g028225
MrUN20695	Medtr3g020780
MrUN20767	Medtr4g024370
MrUN20859	Medtr8g023060
MrUN20860	Medtr8g023060
MrUN20893	Medtr8g064180
MrUN20894	Medtr3g070860
MrUN20896	Medtr5g094450
MrUN20899	Medtr7g012260
MrUN21000	Medtr3g090900
MrUN21009	Medtr1g013700
MrUN21014	Medtr3g045790
MrUN21045	Medtr3g112530
MrUN21065	Medtr7g417750
MrUN21068	Medtr4g052010
MrUN21111	Medtr3g094080

Medicago ruthenica transcripts	*Medicago truncatula* genes
MrUN21126	Medtr3g116380
MrUN21198	Medtr8g100120
MrUN21215	Medtr5g090780
MrUN21238	Medtr5g017210
MrUN21246	Medtr3g491890
MrUN21286	Medtr4g094275
MrUN21288	Medtr2g105090
MrUN21336	Medtr3g093780
MrUN21340	Medtr7g013820
MrUN21389	Medtr2g082410
MrUN21406	Medtr7g023290
MrUN21482	Medtr2g082580
MrUN21528	Medtr1g077790
MrUN21550	Medtr7g011850
MrUN21568	Medtr5g041910
MrUN21597	Medtr2g013140
MrUN21604	Medtr4g107010
MrUN21775	Medtr2g082930
MrUN21777	Medtr7g085490
MrUN21973	Medtr3g064700
MrUN22008	Medtr3g101670
MrUN22052	Medtr2g041550
MrUN22135	Medtr4g109310
MrUN22378	Medtr6g065460
MrUN22386	Medtr2g020060
MrUN22417	Medtr2g020160
MrUN22422	Medtr5g064640
MrUN22480	Medtr8g021380
MrUN22492	Medtr4g102660
MrUN22499	Medtr3g078800
MrUN22508	Medtr4g010140
MrUN22519	Medtr2g436310
MrUN22531	Medtr7g091280
MrUN22554	Medtr5g005420
MrUN22559	Medtr4g085890
MrUN22588	Medtr3g103960
MrUN22623	Medtr5g029370
MrUN22631	Medtr4g129670
MrUN22647	Medtr2g042720
MrUN22721	Medtr8g073120
MrUN22735	Medtr5g020940
MrUN22745	Medtr8g093440
MrUN22776	Medtr8g099135
MrUN22808	Medtr4g088170

Medicago ruthenica transcripts	*Medicago truncatula* genes
MrUN22829	Medtr7g099820
MrUN22836	Medtr4g007080
MrUN22866	Medtr1g059970
MrUN22909	Medtr8g032260
MrUN22952	Medtr4g015650
MrUN22964	Medtr7g009820
MrUN22983	Medtr8g017100
MrUN23007	Medtr8g099665
MrUN23013	Medtr7g080530
MrUN23022	Medtr2g078620
MrUN23024	Medtr4g085990
MrUN23040	Medtr1g080990
MrUN23068	Medtr8g107010
MrUN23070	Medtr3g095340
MrUN23103	Medtr7g085200
MrUN23131	Medtr4g086020
MrUN23137	Medtr5g011990
MrUN23150	Medtr4g129560
MrUN23186	Medtr3g105100
MrUN23217	Medtr3g463720
MrUN23224	Medtr4g079160
MrUN23253	Medtr2g084020
MrUN23254	Medtr1g006490
MrUN23266	Medtr8g096640
MrUN23297	Medtr2g010520
MrUN23338	Medtr5g045300
MrUN23343	Medtr4g054920
MrUN23373	Medtr4g133620
MrUN23391	Medtr5g012000
MrUN23392	Medtr4g121570
MrUN23409	Medtr4g089080
MrUN23418	Medtr7g111860
MrUN23423	Medtr4g099510
MrUN23424	Medtr7g405770
MrUN23645	Medtr5g084790
MrUN23666	Medtr5g023730
MrUN23809	Medtr1g084790
MrUN24189	Medtr2g096670
MrUN24239	Medtr7g102450
MrUN24271	Medtr3g082830
MrUN24353	Medtr5g058090
MrUN24614	Medtr2g089120
MrUN24848	Medtr1g061540
MrUN24881	Medtr2g067640

Medicago ruthenica transcripts	*Medicago truncatula* genes
MrUN24901	Medtr6g012520
MrUN24906	Medtr3g084520
MrUN25469	Medtr7g032900
MrUN25647	Medtr6g012830
MrUN25897	Medtr1g097270
MrUN26033	Medtr8g077590
MrUN26102	Medtr4g035180
MrUN26149	Medtr1g076940
MrUN26440	Medtr2g073560
MrUN26610	Medtr3g462340
MrUN26654	Medtr7g073380
MrUN26815	Medtr5g025610
MrUN27006	Medtr3g072300
MrUN27032	Medtr2g011210
MrUN27227	Medtr5g087360
MrUN27235	Medtr5g031360
MrUN27265	Medtr7g118300
MrUN27439	Medtr7g076900
MrUN27442	Medtr4g124790
MrUN27551	Medtr2g029910
MrUN27626	Medtr4g015630
MrUN27630	Medtr1g101550
MrUN27658	Medtr7g093040
MrUN27662	Medtr3g113970
MrUN27747	Medtr4g088770
MrUN27802	Medtr5g024020
MrUN27969	Medtr6g032880
MrUN28008	Medtr7g011010
MrUN28030	Medtr4g103330
MrUN28096	Medtr3g114120
MrUN28151	Medtr4g076640
MrUN28309	Medtr6g033725
MrUN28356	Medtr2g011080
MrUN28509	Medtr3g098910
MrUN28528	Medtr2g084805
MrUN28677	Medtr7g100240
MrUN28730	Medtr7g009930
MrUN28808	Medtr5g011220
MrUN28830	Medtr6g038220
MrUN28870	Medtr3g437870
MrUN29644	Medtr2g064930
MrUN29706	Medtr3g464580
MrUN29740	Medtr2g049020
MrUN29850	Medtr4g122180

Medicago ruthenica transcripts	*Medicago truncatula* genes
MrUN30284	Medtr4g017640
MrUN30337	Medtr8g024380
MrUN30731	Medtr2g090200
MrUN30810	Medtr0280s0040
MrUN30812	Medtr5g007713
MrUN30814	Medtr7g101395
MrUN30828	Medtr4g109450
MrUN31036	Medtr6g016820
MrUN31173	Medtr2g034480
MrUN31229	Medtr5g089750
MrUN31657	Medtr3g099200
MrUN31680	Medtr1g006600
MrUN31749	Medtr1g090873
MrUN31811	Medtr1g110930
MrUN31815	Medtr6g090380
MrUN31820	Medtr5g064610
MrUN31873	Medtr3g088970
MrUN31898	Medtr5g014090
MrUN31937	Medtr4g063710
MrUN31952	Medtr8g039190
MrUN31965	Medtr2g095130
MrUN31975	Medtr8g016030
MrUN32009	Medtr8g464570
MrUN32030	Medtr4g109170
MrUN32034	Medtr1g061050
MrUN32062	Medtr7g018170
MrUN32093	Medtr2g049020
MrUN32104	Medtr6g016875
MrUN32119	Medtr3g010770
MrUN32126	Medtr4g070430
MrUN32253	Medtr4g130540
MrUN32343	Medtr5g030890
MrUN32350	Medtr2g079650
MrUN32475	Medtr1g077000
MrUN32628	Medtr8g054450
MrUN32664	Medtr5g020990
MrUN32715	Medtr8g006730
MrUN32724	Medtr5g017470
MrUN32747	Medtr7g024320
MrUN32817	Medtr4g125940
MrUN32882	Medtr5g070330
MrUN32965	Medtr3g498825
MrUN32999	Medtr1g063850
MrUN33003	Medtr4g116420

Medicago ruthenica transcripts	*Medicago truncatula* genes
MrUN33021	Medtr0154s0040
MrUN33047	Medtr8g033290
MrUN33067	Medtr8g020920
MrUN33075	Medtr5g098420
MrUN33159	Medtr2g097350
MrUN33180	Medtr3g060240
MrUN33211	Medtr5g022970
MrUN33257	Medtr2g089795
MrUN33270	Medtr6g007807
MrUN33296	Medtr4g079850
MrUN33341	Medtr8g087890
MrUN33367	Medtr7g093820
MrUN33405	Medtr4g128570
MrUN33413	Medtr6g004430
MrUN33474	Medtr6g078450
MrUN33504	Medtr7g096930
MrUN33528	Medtr5g464340
MrUN33555	Medtr4g098620
MrUN33740	Medtr1g011800
MrUN33820	Medtr5g020570
MrUN33864	Medtr3g098500
MrUN33885	Medtr2g007960
MrUN33893	Medtr5g031250
MrUN33925	Medtr4g121080
MrUN33935	Medtr3g062890
MrUN33992	Medtr6g043240
MrUN34038	Medtr4g082060
MrUN34050	Medtr1g087540
MrUN34082	Medtr6g033675
MrUN34094	Medtr7g072510
MrUN34136	Medtr4g099130
MrUN34143	Medtr4g133230
MrUN34180	Medtr5g018570
MrUN34198	Medtr4g019210
MrUN34220	Medtr4g066010
MrUN34262	Medtr7g116520
MrUN34292	Medtr6g088670
MrUN34388	Medtr1g034120
MrUN34411	Medtr3g088110
MrUN34441	Medtr8g466240
MrUN34447	Medtr5g082130
MrUN34467	Medtr4g107970
MrUN34482	Medtr6g015815
MrUN34486	Medtr2g013780

Medicago ruthenica transcripts	*Medicago truncatula* genes
MrUN34492	Medtr6g023910
MrUN34535	Medtr1g038810
MrUN34664	Medtr8g015950
MrUN34681	Medtr3g116770
MrUN34813	Medtr4g073690
MrUN34821	Medtr5g048160
MrUN34827	Medtr8g099685
MrUN34887	Medtr5g098020
MrUN34920	Medtr1g022445
MrUN34936	Medtr1g059960
MrUN34950	Medtr2g034470
MrUN34951	Medtr2g034470
MrUN34957	Medtr4g011790
MrUN35002	Medtr3g101400
MrUN35074	Medtr8g089840
MrUN35078	Medtr5g056480
MrUN35109	Medtr1g115210
MrUN35113	Medtr6g088670
MrUN35194	Medtr4g037670
MrUN35214	Medtr4g015010
MrUN35391	Medtr2g022480
MrUN35408	Medtr5g096150
MrUN35415	Medtr1g101790
MrUN35533	Medtr7g065890
MrUN35562	Medtr7g068190
MrUN35574	Medtr4g025730
MrUN35703	Medtr6g008560
MrUN35766	Medtr5g030500
MrUN35833	Medtr0443s0040
MrUN35863	Medtr4g114960
MrUN35925	Medtr4g057200
MrUN35961	Medtr8g079950
MrUN35975	Medtr8g068890
MrUN35979	Medtr8g096920
MrUN35988	Medtr3g094000
MrUN36001	Medtr1g080110
MrUN36009	Medtr3g105550
MrUN36032	Medtr4g104700
MrUN36160	Medtr1g052640
MrUN36188	Medtr7g093450
MrUN36220	Medtr7g116500
MrUN36251	Medtr2g027780
MrUN36403	Medtr8g432470
MrUN36404	Medtr8g432490

Medicago ruthenica transcripts	*Medicago truncatula* genes
MrUN36406	Medtr7g081270
MrUN36420	Medtr8g104520
MrUN36429	Medtr6g012450
MrUN36459	Medtr5g048550
MrUN36477	Medtr3g024510
MrUN36661	Medtr1g026410
MrUN36773	Medtr4g131400
MrUN36775	Medtr5g096670
MrUN36810	Medtr1g107380
MrUN36812	Medtr8g027745
MrUN36837	Medtr3g081500
MrUN36911	Medtr4g130540
MrUN36942	Medtr2g088770
MrUN36951	Medtr3g101560
MrUN36998	Medtr3g118290
MrUN37008	Medtr8g009080
MrUN37015	Medtr1g040430
MrUN37095	Medtr6g016640
MrUN37165	Medtr5g042420
MrUN37204	Medtr6g069560
MrUN37264	Medtr6g472100
MrUN37384	Medtr5g017850
MrUN37393	Medtr5g017850
MrUN37397	Medtr3g078700
MrUN37404	Medtr5g012290
MrUN37456	Medtr2g099010
MrUN37511	Medtr7g114900
MrUN37527	Medtr7g458880
MrUN37532	Medtr4g086620
MrUN37540	Medtr3g104980
MrUN37588	Medtr3g101290
MrUN37703	Medtr8g081620
MrUN37754	Medtr7g105870
MrUN37773	Medtr1g090290
MrUN37796	Medtr5g090580
MrUN37838	Medtr8g090205
MrUN37885	Medtr7g114590
MrUN38006	Medtr2g079990
MrUN38027	Medtr7g092460
MrUN38163	Medtr7g074250
MrUN38198	Medtr3g479470
MrUN38295	Medtr1g115740
MrUN38328	Medtr8g024050
MrUN38351	Medtr4g094625

Medicago ruthenica transcripts	*Medicago truncatula* genes
MrUN38397	Medtr5g059820
MrUN38400	Medtr1g084790
MrUN38409	Medtr8g019660
MrUN38500	Medtr8g092460
MrUN38544	Medtr7g117310
MrUN38556	Medtr2g098250
MrUN38576	Medtr8g090025
MrUN38635	Medtr8g071130
MrUN38728	Medtr7g088330
MrUN38821	Medtr7g086690
MrUN38852	Medtr4g086190
MrUN38853	Medtr8g018620
MrUN38888	Medtr8g020590
MrUN38955	Medtr2g100340
MrUN38960	Medtr8g085900
MrUN38966	Medtr7g084770
MrUN39089	Medtr7g024390
MrUN39307	Medtr8g070830
MrUN39353	Medtr6g088475
MrUN39354	Medtr6g088445
MrUN39357	Medtr2g022700
MrUN39360	Medtr5g025650
MrUN39410	Medtr5g041700
MrUN39435	Medtr7g074710
MrUN39474	Medtr8g014910
MrUN39493	Medtr4g128260
MrUN39518	Medtr3g090760
MrUN39528	Medtr0184s0030
MrUN39534	Medtr3g091760
MrUN39573	Medtr0072s0040
MrUN39580	Medtr6g069600
MrUN39618	Medtr4g065077
MrUN39673	Medtr4g118480
MrUN39737	Medtr2g089140
MrUN39798	Medtr0035s0150
MrUN39857	Medtr1g007380
MrUN39986	Medtr4g087160
MrUN40033	Medtr1g100623
MrUN40041	Medtr7g106000
MrUN40064	Medtr3g082100
MrUN40162	Medtr7g097060
MrUN40182	Medtr7g118330
MrUN40196	Medtr3g091340
MrUN40259	Medtr3g112160

Medicago ruthenica transcripts	*Medicago truncatula* genes
MrUN40292	Medtr8g098410
MrUN40317	Medtr7g106340
MrUN40324	Medtr4g127290
MrUN40347	Medtr5g012810
MrUN40409	Medtr2g450080
MrUN40421	Medtr1g016070
MrUN40510	Medtr6g090460
MrUN40571	Medtr1g086650
MrUN40603	Medtr2g099950
MrUN40627	Medtr5g011250
MrUN40629	Medtr8g026960
MrUN40647	Medtr3g071740
MrUN40658	Medtr7g114490
MrUN40668	Medtr4g021800
MrUN40670	Medtr5g094390
MrUN40683	Medtr7g114560
MrUN40762	Medtr3g110450
MrUN40811	Medtr1g110870
MrUN40859	Medtr7g084300
MrUN40889	Medtr8g042870
MrUN40897	Medtr6g023340
MrUN40911	Medtr2g044920
MrUN41186	Medtr4g119780
MrUN41238	Medtr4g009620
MrUN41386	Medtr7g093260
MrUN41525	Medtr1g057270
MrUN41531	Medtr7g068770
MrUN41628	Medtr1g116120
MrUN41710	Medtr8g081040
MrUN41763	Medtr1g035690
MrUN41921	Medtr5g014520
MrUN42177	Medtr5g039390
MrUN42287	Medtr4g094010
MrUN42510	Medtr7g088820
MrUN42784	Medtr7g116510
MrUN42792	Medtr1g031650
MrUN42817	Medtr2g087950
MrUN42898	Medtr4g077070
MrUN42900	Medtr1g077990
MrUN42944	Medtr2g040000
MrUN42987	Medtr3g064080
MrUN43048	Medtr8g069825
MrUN43119	Medtr4g122500
MrUN43148	Medtr5g019010

Medicago ruthenica transcripts	*Medicago truncatula* genes
MrUN43224	Medtr3g074230
MrUN43417	Medtr5g029750
MrUN43425	Medtr3g065440
MrUN43624	Medtr7g111030
MrUN43687	Medtr5g098170
MrUN43708	Medtr4g094828
MrUN43724	Medtr7g101930
MrUN43733	Medtr5g011520
MrUN43751	Medtr5g036410
MrUN43784	Medtr1g102360
MrUN43867	Medtr6g009200
MrUN43957	Medtr2g437380
MrUN43958	Medtr4g077620
MrUN44014	Medtr2g090960
MrUN44068	Medtr2g045780
MrUN44105	Medtr3g009010
MrUN44212	Medtr3g008010
MrUN44305	Medtr2g100560
MrUN44465	Medtr7g062580
MrUN44499	Medtr7g072605
MrUN44771	Medtr5g030920
MrUN44849	Medtr5g026500
MrUN44880	Medtr3g070800
MrUN44965	Medtr3g437630
MrUN45155	Medtr7g085310
MrUN45232	Medtr1g073740
MrUN45424	Medtr7g098650
MrUN45431	Medtr8g022810
MrUN45489	Medtr2g011180
MrUN56078	Medtr7g050980
MrUN59732	Medtr1g019750
MrUN60721	Medtr1g115535
MrUN62562	Medtr4g009590
MrUN63427	Medtr0056s0160
MrUN63913	Medtr4g092550
MrUN63915	Medtr4g092530
MrUN64793	Medtr1g105860
MrUN65459	Medtr5g013640
MrUN65610	Medtr1g111100
MrUN66246	Medtr1g038430
MrUN68267	Medtr2g034720
MrUN68297	Medtr8g045890
MrUN68514	Medtr2g021190
MrUN69372	Medtr2g084020

Medicago ruthenica transcripts	*Medicago truncatula* genes
MrUN69802	Medtr4g108100
MrUN71337	Medtr4g097920
MrUN72843	Medtr8g032000
MrUN72967	Medtr4g033325
MrUN74016	Medtr1g090687
MrUN74117	Medtr6g048440
