# Supplementary material for: Transcriptome sequencing and expression profiling of genes involved in the response to abiotic stress in Medicago ruthenica
Source: Genet Mol Biol. 2018 Jun 28;41(3):638–48. doi: 10.1590/1678-4685-GMB-2017-0284 (PMC6136363; doi:10.1590/1678-4685-GMB-2017-0284)
Supplement: Supplementary file 6 [file 1415-4757-GMB-1678-4685-GMB-2017-0284-s007.pdf]

Supplementary Material to "Transcriptome sequencing and expression profiling of genes involved in the response to abiotic stress in *Medicago ruthenica*"

Table S4 - Functional enrichment analysis of GO terms using topGO for genes in the *Medicago ruthenica* response to abiotic stress.

Group	GO Terms	Term Description	Total Transcripts	Significant Transcripts	*P*-value
	GO:0046906	tetrapy rrole binding	650	32	8.50E-09
	GO:0016209	antioxidant activity	207	17	2.30E-08
	GO:0004601	peroxidase activity	183	16	2.50E-08
	GO:0016684	oxidoreductase activity, acting on perox...	183	16	2.50E-08
	GO:0020037	heme binding	621	30	3.80E-08
	GO:0016757	transferase activity, transferring glyco...	1001	40	4.00E-08
	GO:0016758	transferase activity, transferring hexos...	771	34	4.30E-08
	GO:0016491	oxidoreductase activity	2767	79	5.40E-08
	GO:0016762	xyloglucan:xyloglucosyl transferase acti...	63	8	5.00E-06
	GO:0009055	electron carrier activity	189	12	3.60E-05
	GO:0003824	catalytic activity	16955	305	6.00E-05
**MF**	GO:0004553	hydrolase activity, hydrolyzing O-glycos...	822	26	0.00042
	GO:0016798	hydrolase activity, acting on glycosyl b...	883	26	0.00119
	GO:0000990	core RNA polymerase binding transcriptio...	15	3	0.00139
	GO:0000996	core DNA-dependent RNA polymerase bindin...	15	3	0.00139
	GO:0016987	sigma factor activity	15	3	0.00139
	GO:0005507	copper ion binding	184	9	0.00213
	GO:0004014	adenosylmethionine decarboxylase activit...	5	2	0.00224
	GO:0015035	protein disulfide oxidoreductase activit...	61	5	0.00235
	GO:0015036	disulfide oxidoreductase activity	61	5	0.00235
	GO:0030599	pectinesterase activity	96	6	0.00351

Group	GO Terms	Term Description	Total Transcripts	Significant Transcripts	*P*-value
	GO:0001071	nucleic acid binding transcription facto...	1372	34	0.004
	GO:0003700	sequence-specific DNA binding transcript...	1372	34	0.004
	GO:0016746	transferase activity, transferring acyl ...	544	17	0.00447
	GO:0016759	cellulose synthase activity	71	5	0.00455
	GO:0016760	cellulose synthase (UDP-forming) activit...	71	5	0.00455
	GO:0004332	fructose-bisphosphate aldolase activity	8	2	0.00607
	GO:0016705	oxidoreductase activity, acting on paire...	854	23	0.00647
	GO:0008171	O-methyltransferase activity	78	5	0.00677
	GO:0016706	oxidoreductase activity, acting on paire...	307	11	0.00781
	GO:0016614	oxidoreductase activity, acting on CH-OH...	308	11	0.008
	GO:0052689	carboxylic ester hydrolase activity	227	9	0.00831
	GO:0051213	dioxygenase activity	403	13	0.00939
BP	GO:0015979	photosynthesis	148	16	4.50E-09
	GO:0055114	oxidation-reduction process	2442	77	6.40E-08
	GO:0006979	response to oxidative stress	282	19	3.70E-07
	GO:0006073	cellular glucan metabolic process	205	15	2.20E-06
	GO:0044042	glucan metabolic process	205	15	2.20E-06
	GO:0005976	polysaccharide metabolic process	267	17	3.20E-06
	GO:0019684	photosynthesis, light reaction	58	8	5.50E-06
	GO:0009767	photosynthetic electron transport chain	17	5	6.80E-06
	GO:0006950	response to stress	1697	53	1.10E-05
	GO:0044264	cellular polysaccharide metabolic proces...	241	15	1.60E-05
	GO:0009773	photosynthetic electron transport in pho...	11	4	2.40E-05
	GO:0051607	defense response to virus	13	4	5.00E-05
	GO:0006952	defense response	328	16	0.00015
	GO:0005975	carbohydrate metabolic process	1522	45	0.00019
	GO:0002252	immune effector process	19	4	0.00025
	GO:0022900	electron transport chain	55	6	0.00031

Group	GO Terms	Term Description	Total Transcripts	Significant Transcripts	*P*-value
	GO:0044710	single-organism metabolic process	5672	126	0.0004
	GO:0044262	cellular carbohydrate metabolic process	400	17	0.00049
	GO:0008152	metabolic process	17713	334	0.00061
	GO:0002376	immune system process	113	8	0.00065
	GO:0006091	generation of precursor metabolites and ...	240	12	0.00081
	GO:0009607	response to biotic stimulus	352	15	0.001
	GO:0098542	defense response to other organism	157	9	0.00139
	GO:0042545	cell wall modification	129	8	0.00155
	GO:0009615	response to virus	77	6	0.00188
	GO:0071555	cell wall organization	166	9	0.00205
	GO:0019318	hexose metabolic process	167	9	0.00213
	GO:0043207	response to external biotic stimulus	309	13	0.00236
	GO:0051707	response to other organism	309	13	0.00236
	GO:0045229	external encapsulating structure organiz...	172	9	0.0026
	GO:0006597	spermine biosynthetic process	5	2	0.00272
	GO:0008215	spermine metabolic process	5	2	0.00272
	GO:0008216	spermidine metabolic process	5	2	0.00272
	GO:0008295	spermidine biosynthetic process	5	2	0.00272
	GO:0030244	cellulose biosynthetic process	90	6	0.00412
	GO:0010150	leaf senescence	20	3	0.00433
	GO:0010260	organ senescence	20	3	0.00433
	GO:0071554	cell wall organization or biogenesis	257	11	0.00436
	GO:0030243	cellulose metabolic process	93	6	0.00484
	GO:0009699	phenylpropanoid biosynthetic process	21	3	0.00499
	GO:0009806	lignan metabolic process	21	3	0.00499
	GO:0009807	lignan biosynthetic process	21	3	0.00499
	GO:0016052	carbohydrate catabolic process	159	8	0.00558
	GO:0006595	polyamine metabolic process	7	2	0.00558
	GO:0006596	polyamine biosynthetic process	7	2	0.00558
	GO:0033993	response to lipid	194	9	0.00572
	GO:0009250	glucan biosynthetic process	134	7	0.00761
	GO:0006006	glucose metabolic process	136	7	0.00823

Group	GO Terms	Term Description	Total Transcripts	Significant Transcripts	*P*-value
	GO:0009698	phenylpropanoid metabolic process	26	3	0.00917
	GO:0048589	developmental growth	108	6	0.00986
	GO:0044436	thylakoid part	330	28	7.50E-13
	GO:0009579	thylakoid	358	29	9.20E-13
	GO:0042651	thylakoid membrane	262	25	1.00E-12
	GO:0034357	photosynthetic membrane	300	26	3.20E-12
	GO:0005618	cell wall	355	27	2.50E-11
	GO:0030312	external encapsulating structure	355	27	2.50E-11
	GO:0009534	chloroplast thylakoid	237	20	1.80E-09
	GO:0031976	plastid thylakoid	237	20	1.80E-09
	GO:0031984	organelle subcompartment	237	20	1.80E-09
	GO:0009535	chloroplast thylakoid membrane	207	18	7.30E-09
	GO:0055035	plastid thylakoid membrane	207	18	7.30E-09
	GO:0044434	chloroplast part	603	31	1.30E-08
	GO:0044435	plastid part	630	31	3.50E-08
	GO:0009523	photosystem II	55	9	2.10E-07
	GO:0009507	chloroplast	2129	65	2.20E-07
	GO:0009536	plastid	2187	65	5.90E-07
**CC**	GO:0009654	photosystem II oxygen evolving complex	35	7	1.20E-06
	GO:0009521	photosystem	88	10	1.50E-06
	GO:0048046	apoplast	128	11	7.20E-06
	GO:0019898	extrinsic component of membrane	50	7	1.40E-05
	GO:0005576	extracellular region	221	14	1.50E-05
	GO:0009505	plant-type cell wall	81	8	4.80E-05
	GO:1990204	oxidoreductase complex	96	8	0.00016
	GO:0016020	membrane	5642	120	0.00018
	GO:0071944	cell periphery	1496	42	0.00028
	GO:0009570	chloroplast stroma	226	12	0.00031
	GO:0009543	chloroplast thylakoid lumen	41	5	0.00049
	GO:0031978	plastid thylakoid lumen	41	5	0.00049
	GO:0009532	plastid stroma	255	12	0.00092
	GO:0031977	thylakoid lumen	57	5	0.00221
	GO:0010598	NAD(P)H dehydrogenase complex (plastoqui...	5	2	0.0025
	GO:0031012	extracellular matrix	21	3	0.00443
