# Supplementary material for: Transcriptome sequencing and expression profiling of genes involved in the response to abiotic stress in Medicago ruthenica
Source: Genet Mol Biol. 2018 Jun 28;41(3):638–48. doi: 10.1590/1678-4685-GMB-2017-0284 (PMC6136363; doi:10.1590/1678-4685-GMB-2017-0284)
Supplement: Supplementary file 7 [file 1415-4757-GMB-1678-4685-GMB-2017-0284-s003.pdf]

Supplementary Material to "Transcriptome sequencing and expression profiling of genes involved in the response to abiotic stress in *Medicago ruthenica*"

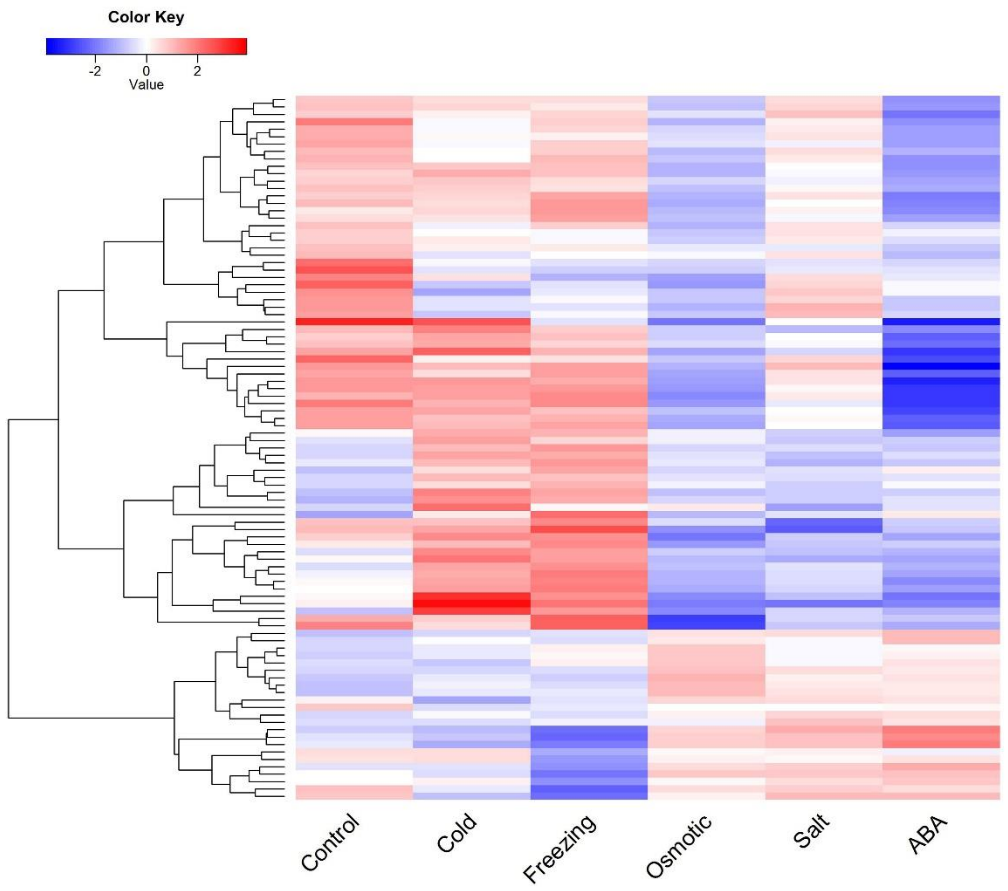

Figure S3 - Heatmap showing the expression profiles of transcription factor genes that are differentially expressed in response to abiotic stress in *Medicago ruthenica*. The heatmap was generated using the R gplots package, the FPKM values for each transcript were normalized and used as input; red represents high levels of expression, while blue represents low expression levels.
