# Supplementary material for: Transcriptome sequencing and expression profiling of genes involved in the response to abiotic stress in Medicago ruthenica
Source: Genet Mol Biol. 2018 Jun 28;41(3):638–48. doi: 10.1590/1678-4685-GMB-2017-0284 (PMC6136363; doi:10.1590/1678-4685-GMB-2017-0284)
Supplement: Supplementary file 8 [file 1415-4757-GMB-1678-4685-GMB-2017-0284-s008.pdf]

Supplementary Material to "Transcriptome sequencing and expression profiling of genes involved in the response to abiotic stress in *Medicago ruthenica*"

Table S5 - Phytohormone-related unigenes that were differentially expressed in response to abiotic stress in *Medicago ruthenica*.

Hormone	UniGenes	Log2(Fold Changes)				
		Cold	Freezing	Osmotic	Salt	ABA
ABA	MrUN01823	3.08	2.77	2.23	0.79	4.44
	MrUN02488	-1.61	-0.63	1.67	1.61	1.32
	MrUN03429	-2.07	-2.33	-1.24	0.13	-0.82
	MrUN04931	0.73	-4.97	1.31	1.87	1.88
	MrUN12110	-0.52	-3.37	-0.64	0.65	0.55
	MrUN19623	2.61	-1.59	1.69	-0.35	2.83
	MrUN20299	0.13	-3.30	0.79	1.71	1.82
	MrUN27218	-0.88	0.00	0.20	2.79	-1.84
	MrUN31898	-0.05	0.51	-2.22	-1.41	-2.37
	MrUN32485	-0.11	0.62	-1.92	-0.38	-2.70
	MrUN33922	1.47	1.15	-0.54	-0.64	-0.13
	MrUN35766	1.53	1.39	-0.78	-0.54	-0.54
	MrUN38104	-0.12	-1.50	0.02	1.40	-0.04
	MrUN38702	-3.25	-4.75	-3.02	-1.31	-2.09
	MrUN64793	-3.16	-2.41	-1.10	-0.70	-2.46
	MrUN68297	-2.02	-0.99	-2.03	-0.94	-0.17
GA	MrUN05525	-1.17	-2.42	1.20	1.48	2.54
	MrUN07876	0.76	0.96	0.86	0.87	2.04
	MrUN19564	1.46	0.27	1.65	-1.54	1.75
	MrUN21275	2.28	0.88	2.13	2.08	2.28
	MrUN21674	2.64	2.21	0.63	0.12	0.58
	MrUN27354	-0.57	-0.14	1.96	3.01	2.74
	MrUN39273	-0.12	-0.59	0.91	1.52	1.67
Auxin	MrUN02313	4.04	4.45	2.68	2.52	4.34
	MrUN02949	-0.35	0.50	-3.31	-1.35	-3.25
	MrUN09781	2.12	2.01	2.45	1.07	3.41
	MrUN19451	1.95	2.08	1.95	0.86	1.94
	MrUN20456	1.23	-2.74	-1.21	-0.36	-0.53
	MrUN22415	0.21	-0.58	1.89	0.84	1.77
	MrUN25721	0.39	0.92	-1.79	-0.99	-2.15
	MrUN27459	0.19	-3.20	1.76	2.60	3.36

Hormone	UniGenes	Log2(Fold Changes)				
		Cold	Freezing	Osmotic	Salt	ABA
	MrUN30617	-0.38	0.48	-2.71	-1.81	-2.57
	MrUN31229	2.18	2.20	0.18	-0.81	-0.09
	MrUN34160	0.36	0.31	-2.03	-0.18	-1.33
	MrUN36270	-0.62	-2.24	0.15	0.22	0.50
	MrUN37653	4.26	3.47	1.94	2.73	2.61
	MrUN39798	-0.15	0.46	-1.12	-1.08	-2.16
	MrUN41386	2.53	2.40	-0.44	-0.10	0.47
	MrUN42128	-1.68	-3.54	-2.89	-1.52	-1.78
	MrUN45460	-0.58	-7.36	1.71	1.89	3.48
	MrUN63653	-0.40	-1.57	2.41	1.96	3.26
Cytokinin	MrUN33026	-1.06	-4.90	1.73	1.42	2.81
	MrUN01653	0.90	0.68	-2.15	-1.02	-3.51
	MrUN08034	2.65	-0.27	-0.87	-0.63	-1.54
	MrUN22155	1.53	-0.99	1.98	2.24	1.96
JA	MrUN22480	1.76	1.30	-1.20	-1.34	-1.68
	MrUN27819	3.05	-2.16	-2.59	-1.07	-1.44
	MrUN27922	0.13	0.15	-2.92	-1.64	-3.98
	MrUN40276	0.46	0.05	-3.60	-1.78	-3.46
BA	MrUN13345	-0.19	0.03	-3.73	-2.07	-4.27
	MrUN33528	-0.06	0.25	-2.57	-1.71	-2.93
